# Retinotopic remapping of the visual system in deaf adults

**DOI:** 10.1073/pnas.2532413123

**Published:** 2026-09-04

**Authors:** Alexandra T. Levine, Kate Yuen, André Gouws, Alex R. Wade, Antony B. Morland, Charlotte Codina, David Buckley, Heidi A. Baseler

**Affiliations:** ^a^ Department of Psychology, University of York, York YO10 5DD, North Yorkshire, United Kingdom; ^b^ York Neuroimaging Centre, University of York, York YO10 5DD, North Yorkshire, United Kingdom; ^c^ Experimental Medicine and Biomedicine, Hull York Medical School, York YO10 5DD, North Yorkshire, United Kingdom; ^d^ York Biomedical Research Institute, University of York, York North Yorkshire YO10 5DD, United Kingdom; ^e^ Orthoptics & Ophthalmology, School of Allied Health Professions, Pharmacy, Nursing and Midwifery, University of Sheffield, Sheffield S10 2TS, South Yorkshire, United Kingdom

**Keywords:** deaf, cortical plasticity, visual perception, functional MRI, retinotopic mapping

## Abstract

The absence of input from one sense can lead to a greater emphasis on the remaining senses, resulting in heightened performance. Numerous behavioral studies demonstrate that adults who have been deaf from an early age have superior visual sensitivity, particularly to far-peripheral stimuli. We asked whether the increased demand on peripheral vision throughout development that comes with early, profound deafness might be reflected in changes in early visual brain structures. Using functional MRI, we mapped visual field representations in 16 early, profoundly D/deaf adults and 16 hearing age-matched controls. D/deaf individuals exhibited a larger representation of the far-peripheral visual field in both the primary visual cortex and the lateral geniculate nucleus of the thalamus. Importantly, this was not due to a total expansion of the visual map, as there was no difference between groups in overall size of either structure, but a smaller representation of the central visual field in the D/deaf group, suggesting a redistribution of neural resources. Here, we demonstrate that the demands placed on vision due to lifelong deafness can sculpt first level visual map input, to increase neural processing resources for far-peripheral visual stimuli.

## Significance

How are the functions of the brain reorganized when a sensory input is no longer available? Evidence suggests that other senses may compensate; for example, D/deaf adults outperform hearing controls in certain visual tasks. We mapped representations in primary visual cortex and the visual thalamus in the brain. We found that these early visual structures allocated more neural resources to processing peripheral vision relative to central vision in early D/deaf compared to hearing age-matched adults. Our findings suggest that a lifelong greater reliance on vision (rather than hearing in D/deaf adults) to detect and process events in the visual periphery may provoke a redistribution in neuronal resources surprisingly early in neural visual processing.

Sound can serve as a vital cue, helping hearing people orient their gaze and attention toward events outside their central line of sight, especially in the far periphery where vision is typically poor. Without sound cues, deaf people use vision as an “early warning system” for peripheral events; indeed, D/deaf^1^ adults exhibit superior performance in peripheral tasks involving detection or attention to transient or moving targets (1, 2). Many studies report differences in the near periphery (6 to 25°) using a standard visual display (3–6), with some reporting a trade-off in central/peripheral performance (7, 8). In studies testing wider visual fields, the largest effects were found in the far periphery (30 to 85°) for the detection/localization of both transient (9, 10) and moving targets (11, 12).

Previous studies have revealed differences in visually evoked cortical potentials between D/deaf and hearing groups, suggesting potential neural substrates for enhanced visual sensitivity (13–15). More recently, it has been revealed that D/deaf adults recruit auditory regions of the brain in response to visual stimuli (crossmodal plasticity) (10, 16–21) (for reviews, see refs. 22 and 23). Other studies revealed neural differences in D/deaf individuals in higher-order visual areas, such as V5/hMT+ (24), but not in early visual areas (25). However, expanded representations of intact inputs in primary sensory cortex (unimodal plasticity) have been demonstrated in other populations with sensory loss, e.g., amputees (26) and those with early partial sight loss (27, 28). Studies in individuals with early total blindness also demonstrate the potential for plasticity in primary visual cortex. Visual cortex is functionally recruited in blind persons to process nonvisual information obtained from intact sensory modalities (cross-modal plasticity), e.g., tactile (Braille reading) and auditory (spatial localization) processing (29, 30). Further evidence suggests that primary visual cortex demonstrates retinotopic-like responses to spatial sound locations in blind individuals who are expert echolocators (31). Occipital regions may even show plasticity when vision is lost at a later age and enhance the spatial localization of sounds (30, 32). Klinge et al. (33) found clear evidence for stronger corticocortical connections from primary auditory cortex (A1) to V1 in blind adults, which may mediate performance advantages in auditory tasks.

In D/deaf adults, structural changes as early in the visual pathways as the retina have also been correlated with enhanced far-peripheral sensitivity, further supporting the possibility that early visual structures downstream may also be affected (12). Previous studies evaluating primary visual cortex have found either no difference between deaf and hearing groups (25) or thinner visual cortex representing the peripheral visual field (34). Critically, however, these studies were confined to comparisons within a limited visual field (<37.5°), rather than the far periphery where differences in visual sensitivity are most prominent and potentially beneficial (9, 11).

The aim of the current study was to investigate possible neural mechanisms within early visual processing regions in the brain which underpin enhanced peripheral visual sensitivity in D/deaf adults. This was done by mapping wide-field visual representations in early visual structures of D/deaf and hearing adults—the lateral geniculate nucleus (LGN) of the thalamus and primary visual cortex (V1)—to investigate how atypical lifelong sensory experience can reshape visual pathways in the brain. Our data analysis used both participant-specific structural and functional MRI measurements as well as anatomical atlases to evaluate visual representations within the early visual structures. Drawing on the distinct merits of these different approaches, we converged on the same conclusions, namely, a redistribution toward a relatively greater representation of the visual periphery in D/deaf compared to hearing groups.

## Results

**Fig. 1.**
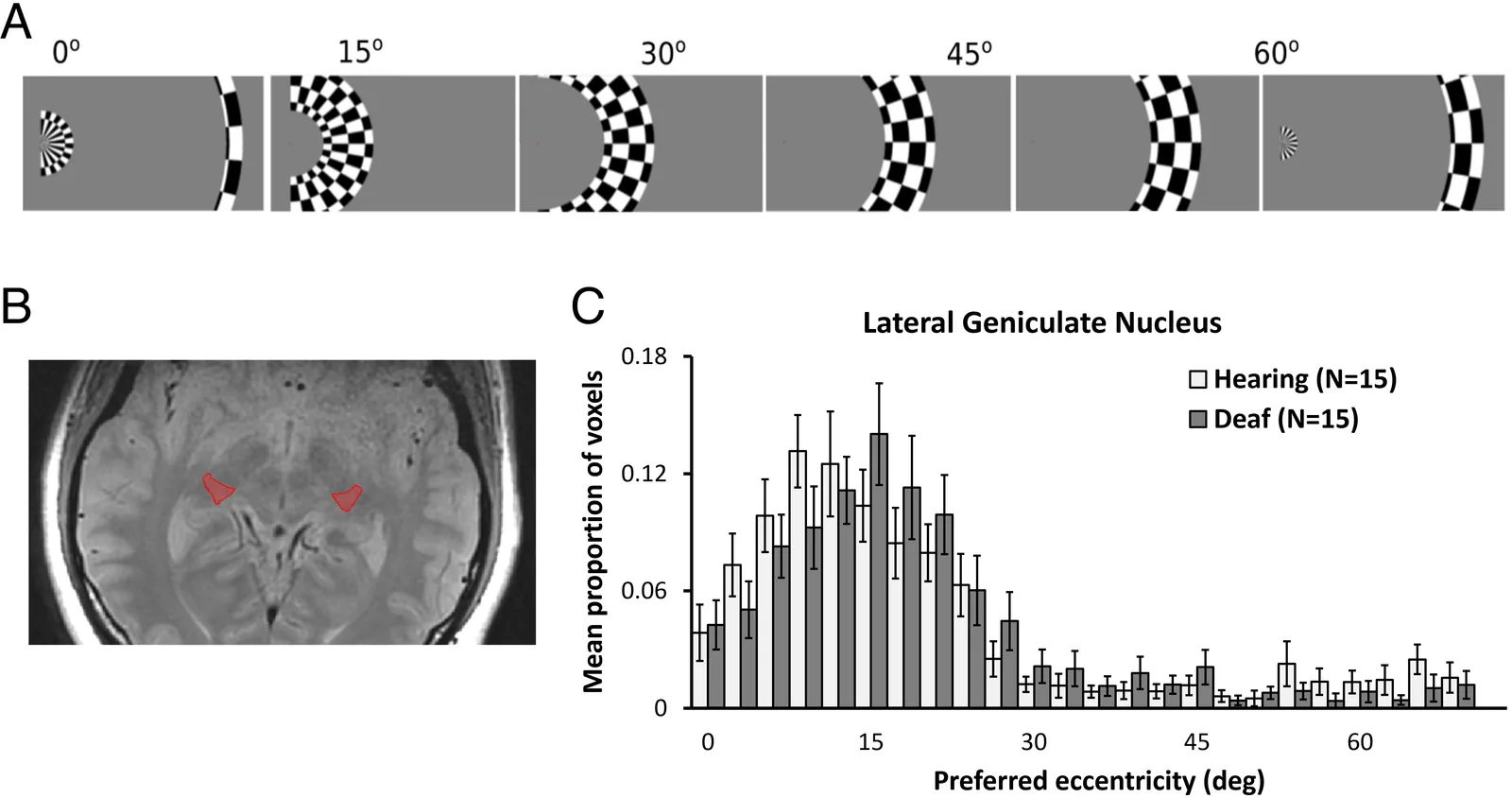
Effects of deafness on the distribution of visual field eccentricity preferences in the LGN. (*A*) Example screenshots of the expanding ring stimulus with contrast-reversing checkerboard in the right hemifield used to measure eccentricity preferences from 0 to 72°. The stimulus at 72° includes a very central ring, and the stimulus at 0° includes a far-peripheral ring, as the stimulus presentation is phase encoded and continuous, and wraps around from central to peripheral presentations. (*B*) Cropped single axial slice of a proton density scan in one participant. Left and right LGN were outlined using anatomical boundaries and are indicated in this slice in red. Further information comparing LGN boundaries defined in the current study with atlas definitions provided in *SI Appendix*, Fig. S1. (*C*) Distributions of eccentricity preference of functionally active voxels in the LGN in hearing (light gray) and deaf (dark gray) groups. Each bar represents the average proportion of voxels functionally activated by the expanding ring stimulus (>0.23 coherence threshold, *P* < 0.01, uncorrected) as a function of approximate preferred eccentricity (divided into 24 bins of 3°). Voxel proportions are cumulative across both hemispheres and averaged across all participants within each group. Error bars represent ± SEM across participants. Eccentricity distributions were significantly different between groups (***P < 0.001). See *Discussion* for an explanation of the apparent “shift” of distribution peaks away from the fovea.

Sixteen adult participants with early, profound deafness (Table 1) and 16 hearing, age-matched controls were recruited to the study. High-resolution T1-weighted structural MR images were also acquired for each participant and were segmented into white and gray matter (cerebral cortex). Using functional MRI, phase-encoded wide-field (72°) retinotopic mapping (Fig. 1*A*) was implemented to map the representation of the visual field within the LGN and primary visual cortex, V1 (35–38). Functional data were carefully aligned to the high-resolution structural images in order to localize and visualize activity on cerebral cortex. The boundaries of the LGN and V1 were defined using each individual participant’s own structural data either anatomically (in the case of the LGN) or using a combination of functionally defined retinotopic borders and anatomical landmarks (in the case of V1). All hand-drawn boundaries were verified by comparison with those fitted using online standard atlases for the human LGN and V1.

**Table 1.** Details of all D/deaf participants in the study

Participant	Age (at testing)	Gender	Handedness	1st Language	BSL^*^	Age of hearing loss (details, if known)
D1	20	F	Left	English	Yes	<3 y (Unknown)
D2	48	F	Right	English	Yes	<3 y (Unknown)
D3	23	F	Right	English	Yes	Birth (Sensorineural loss)
D4	31	M	Left	BSL	Yes	Birth (Hereditary)
D5	31	M	Right	English	Yes	Birth (Unknown)
D6	33	F	Left	BSL	Yes	<3 y (Unknown)
D7	39	M	Right	BSL	Yes	Birth (In utero measles)
D8	20	M	Right	BSL	Yes	Birth (Hereditary)
D9	37	M	Right	English	No	Birth (Birth defect)
D10	20	M	Left	English	No	Birth (Unknown)
D11	43	M	Right	English	Yes	Birth (Hereditary)
D12	48	M	Left	BSL	Yes	Birth (In utero measles)
D13	47	M	Right	English	Yes	Birth (Hereditary)
D14	46	M	Right	English	Yes	Birth (Rubella)
D15	40	M	Right	English	Yes	Birth (Sensorineural loss)
D16	20	F	Right	English	No	2 to 4 y (Unknown)

^*^BSL: British Sign Language

Two different methods were used to quantify the representation of eccentricity from the central to the far peripheral visual field within each brain structure. In the first method, we compared the distribution of eccentricity preferences within the LGN and V1 by calculating the proportion of active voxels within eccentricity bins spread across the visual field. Between-group differences in eccentricity distributions were assessed using both retinotopically defined, hand-drawn boundaries as well as two atlas-defined versions for V1. Once broad agreement was established between the results using three different definitions of V1, we applied a second method to measure the relative volume, surface area, and mean cortical thickness within V1 subregions of interest representing the central, mid-peripheral, and far-peripheral visual field using individual retinotopic data. Evaluating the data using two separate approaches allowed us to mitigate potential subjective bias in hand-drawn boundaries and verify any differences in the visual field representations between groups.

### LGN.

Previous research suggests that projections from peripheral neural retina are greater in D/deaf than hearing adults (12). Therefore, we first assessed whether the redistribution of visual field resources in D/deaf participants might also be detected in the LGN, the thalamic relay structure that receives inputs from the retina and is the main source of projections to V1.

#### Defining the LGN.

Left and right lateral geniculate nuclei were identified anatomically in each participant using structural (proton density) images (Fig. 1*B*) (39). To assess the accuracy of our LGN selection and coregistration, we compared all manually selected LGN boundaries with those defined by the Thalamus Optimized Multi Atlas Segmentation (THOMAS) atlas (40). Although the anatomically defined boundaries of the LGN were generous when accumulated across all participants in standardized MNI space, they overlapped with the THOMAS atlas-defined estimates of the LGN (*SI Appendix*, Fig. S1). Moreover, we verified that there was no overlap of LGN regions with the pulvinar, a nearby subcortical structure that is known to be visually responsive.

#### LGN volume.

Next, we compared the overall size of the LGN between groups. We found that there was no significant difference in the anatomically defined LGN volume between hearing and D/deaf groups (Left + Right LGN combined: Hearing mean = 606.43 mm^3^, SEM, 32.71; D/deaf mean = 608.39 mm^3^, SEM, 34.81; t(28) = 0.041; *P* = 0.968, d = 0.015, CI = −95.88, 99.80). Moreover, the mean volume of functionally active voxels within left and right LGNs combined was in line with previous estimates of human LGN size (41, 42), comprising 150.7 mm^3^ (SEM, 17.7) in the hearing group and 173.7 mm^3^ in the D/deaf group (SEM, 18.3), and there was no significant difference between groups (t(28) = −0.906; *P* = 0.373, d = 0.331, CI = −29.08, 75.21).

#### Visual field representations in the LGN.

As the LGN is a small structure, spatial resolution was not sufficient to generate accurate visual field maps. Therefore, to assess the representation of the visual field in the LGN, we performed a quantitative analysis based purely on functional data. Functional voxels falling within the region of interest were analyzed from expanding ring scans using fast-Fourier transform analysis, where phase of the response represents the preferred retinal eccentricity for each voxel. A coherence threshold of >0.23 (uncorrected *P* < 0.01) was applied to exclude noisy voxels. Histograms representing the distribution of voxels as a function of eccentricity were generated and averaged across participants in each group (Fig. 1*C*). The functionally active voxels within the LGN of each participant were binned according to their preferred eccentricity (stimulus eccentricity producing the maximal functional response). As our phase-encoded stimulus was designed to elicit robust signals across the entire visual field including the far periphery, the distributions did not peak at 0° eccentricity (see *Methodological Considerations* for further explanation). Nevertheless, a two-sample Kolmogorov–Smirnov test demonstrated a significant difference in eccentricity distributions between groups (K = 0.1091, *P* < 0.001; Hearing median = 17°, mode = 11°; D/deaf median = 19°, mode = 15°). Both groups were positively skewed, with the hearing group exhibiting a more positive skew (1.62), indicating a greater preference toward lower (more central) eccentricities compared to the D/deaf group (skew = 1.39). Overall, the LGN data indicate a response bias toward the peripheral visual field in the D/deaf group relative to the hearing group, suggesting that the redistribution of visual field representations can be found in the brain as early as the LGN.

### Primary Visual Cortex (V1).

#### Defining V1.

**Fig. 2.**
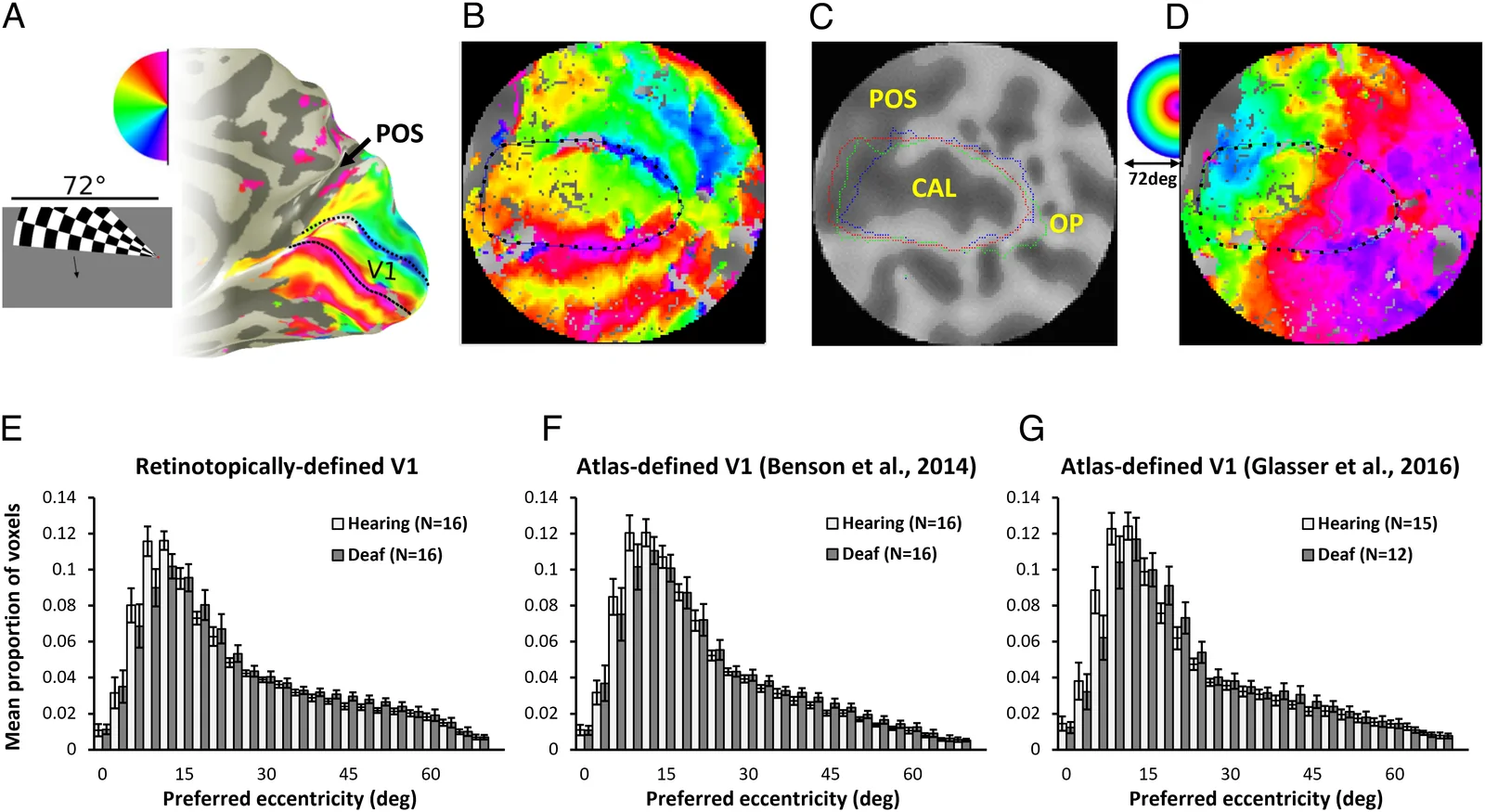
Effects of deafness on the distribution of visual field eccentricity preferences in primary visual cortex (V1). (*A*–*D*) Definition of V1 regions of interest. *Lower Left Inset*: Example of a single frame of the rotating wedge stimulus containing a contrast reversing checkerboard used to map polar angle preferences for the left hemifield (central fixation point shown in red). *Upper Left Inset*: Color map legend denoting polar angle. *Right* panel: Medial view of the right occipital lobe for a single hearing participant (inflated, smoothed 3D surface). Colors indicate preferred polar angle responses of underlying voxels (coherence threshold = 0.20). Color map reversals indicate V1 boundaries at the vertical meridians (dotted black lines). (*B*) The same surface represented as a 2D flat map. Black connected dots indicate hand-drawn boundaries of right V1 at color map reversals. (*C*) The same flattened representation without functional data. Gray scale represents anatomical surface curvature, with light gray denoting gyri and dark gray sulci. Red outline indicates retinotopically guided hand-drawn V1 borders as in (*B*). V1 borders from two different atlases transformed into this individual participant’s structural brain MRI coordinates are shown in blue (43) and green (44). POS: Parieto-occipital sulcus; CAL: Calcarine sulcus; OP: Occipital pole. (*D*) Flattened representation showing preferred eccentricity responses of underlying voxels in the same participant (coherence threshold = 0.20). Color legend on the upper left denotes preferred eccentricity. (*E* and *F*) Distributions of eccentricity preference of functionally active voxels in V1 in hearing (light gray) and deaf (dark gray) groups. Details as in Fig. 1*C*. (*E*) For V1 as defined retinotopically (hand-drawn for each individual). (*F*) For V1 transformed from the atlas in ref. 43. (*G*) For V1 transformed from the Human Connectome Project atlas (HCP_MMP1.0) (44).

Visual area boundaries were defined based on polar angle maps derived from rotating wedge stimulus scans acquired separately from those scans used to map eccentricity representations. Borders were drawn following polar angle reversals on computationally flattened representations of the posterior occipital lobes for each individual and each hemisphere (Fig. 2 *A* and *B* and *SI Appendix*, Fig. S2). All borders were checked by at least two separate observers, one of whom was naïve to the group identity of participants (D/deaf vs. hearing).

Although mapping retinotopic representations from functional MRI data is considered the gold standard for characterizing visual area boundaries in individual participants, it can rely on subjective estimates and border interpolation, particularly where functional retinotopy data are missing or noisy. Therefore, we compared results using V1 boundaries derived from individual retinotopic data in our study to those derived from two publicly available standard human brain atlases using surface-based transforms of V1 boundaries onto individual structural MRI data. Although this carries assumptions about the accuracy of transformation algorithms and whether standard atlas-based visual field maps are appropriate for different study groups, it does allow us a unique opportunity to test these assumptions in a new sample of hearing as well as D/deaf participants. The first was the Benson atlas, which has been validated with retinotopic data within ~0 to 20° eccentricity, but estimates the location and retinotopic organization of V1 from 0 to 90° of eccentricity based on anatomical features (43). The second atlas was that made available by the Human Connectome Project, the HCP_MMP1.0 parcellation (44).

Fig. 2*C* compares V1 boundaries based on individual retinotopy data with those defined using the Benson and HCP atlases on a flattened representation of occipital cortex for a single hearing participant. The regions of interest from the three different definitions of V1 were carefully checked and compared within the T1-weighted structural MRI volume for each participant in each hemisphere using the visualization tool FSLeyes (v.1.9.0; FMRIB Software Library) (45). The position and extent of V1 as defined in our study were a close, qualitative match to those predicted by the Benson and HCP canonical representations of an average V1. Crucially, there was no evidence of systematic bias or difference in the anatomical position of V1 boundaries between D/deaf and hearing groups.

#### Visual field representations in V1.

Next, we calculated distribution of eccentricity preferences of functionally active voxels in V1 defined in three ways, identical to the analysis in the LGN described above (Fig. 2 *E*–*G*). In V1 defined based on individual retinotopic maps (Fig. 2*E*), a two-sample Kolmogorov–Smirnov test revealed a significant difference in distributions between the groups, with a greater preference for more central eccentricities in the hearing group and more peripheral eccentricities in the deaf group (K = 0.0378, *P* < 0.001; Hearing median = 22°, mode = 13°; D/deaf median = 23°, mode = 16°). Both groups were positively skewed, with the hearing group exhibiting a more positive skew (0.84), indicating a greater preference toward lower (more central) eccentricities compared to the D/deaf group (skew = 0.72). This group difference was reproduced using the independent Benson atlas-based definition of V1 transformed on the basis of anatomical features (Fig. 2*F*) (43). A two-sample Kolmogorov–Smirnov test also revealed a significant difference in distributions between the groups, with a greater preference for more central eccentricities in the hearing group and more peripheral eccentricities in the deaf group (K = 0.0414, *P* < 0.001; Hearing median = 20°, mode = 14°, skew = 1.01; D/deaf median = 22°, mode = 14°, skew = 0.87). Finally, the same analysis was applied using the second atlas-based definition of V1 from the Human Connectome Project (Fig. 2*G*) (44). Again, a two-sample Kolmogorov–Smirnov test revealed significant shift in distributions between groups, with the hearing group showing a preference for more central eccentricities and the deaf group for more peripheral eccentricities (K = 0.0781, *P* < 0.001; Hearing median = 20°, mode = 13°, skew = 0.98; D/deaf median = 22°, mode = 15°, skew = 0.80).

### Cortical Measurements Using Retinotopically Defined V1 Subregions.

**Fig. 3.**
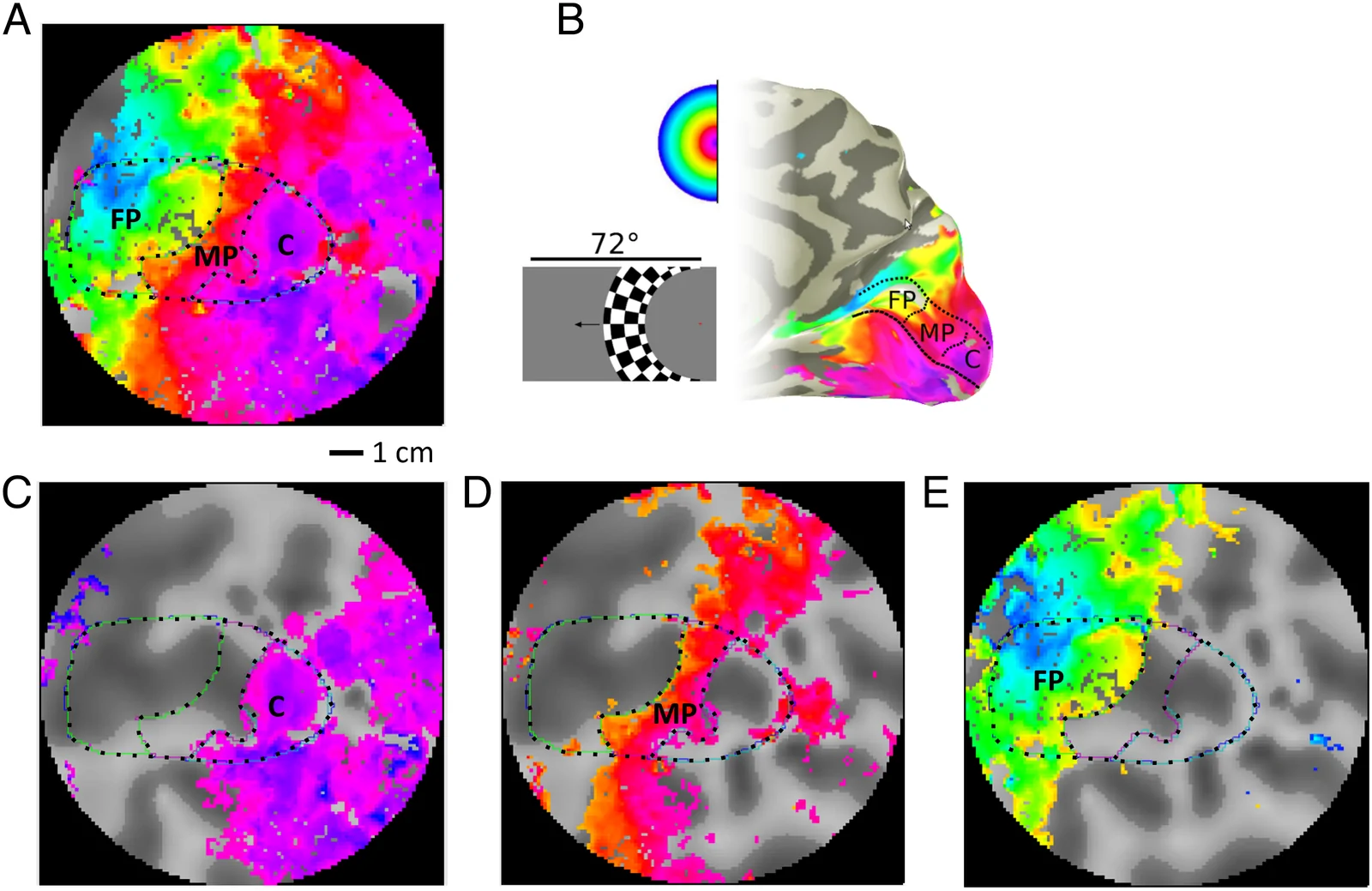
Retinotopic definition of V1 subregions of interest. (*A*) Flattened representation of the right occipital lobe in one hearing participant (coherence threshold = 0.20). Colors represent eccentricity in the left hemifield (color legend shown in *B*). Black dotted lines indicate retinotopically defined V1 borders and boundaries between central (C), mid-peripheral (MP), and far-peripheral (FP) regions of interest. (*B*) *Lower Left Inset*: Example of a single frame of the expanding ring stimulus containing a contrast reversing checkerboard used to map eccentricity preferences for the left hemifield (central fixation point shown in red). *Upper Left Inset*: Color map legend denoting eccentricity. *Right* panel: Medial view of the right occipital lobe for the same hearing participant (inflated, smoothed 3D surface). Colors indicate preferred eccentricity responses of underlying voxels (coherence threshold = 0.20). (*C*) Flat map as in panel *A*, restricted to a phase window representing 0 to 15° to aid manual drawing of the central region of interest, C (coherence threshold = 0.01). (*D*) Flat map as in panel *A*, restricted to a phase window representing 15 to 39° to aid manual drawing of the mid-peripheral region of interest, MP (coherence threshold = 0.01). (*E*) Flat map as in panel *A*, restricted to a phase window representing 39 to 72° to aid manual drawing of the far-peripheral region of interest, FP (coherence threshold = 0.01). This process was repeated in each hemisphere for each participant based on responses to contralateral hemifield stimulation.

Next, we compared structural metrics within primary visual cortex between D/deaf and hearing groups. Currently most human atlases for visual regions are based on retinotopically defined boundaries that do not extend as far into the periphery as measured in our study. Furthermore, current atlases are based on neurotypical individuals and may not represent features unique to the D/deaf population. For this reason, hand-drawn boundaries for specific individuals based on functionally derived retinotopic data were used in the following measurements to avoid these underlying assumptions. Using computationally flattened representations of the posterior occipital lobes in each participant, V1 was divided into three subregions of interest selected to contain roughly the same number of voxels representing three divisions of the visual field: central (0 to 15°); mid-peripheral (15 to 39°) and far-peripheral (39 to 72°) (Fig. 3 and *SI Appendix*, Fig. S2). Importantly, these definitions included a region corresponding to the far periphery (>39°) where previous studies found the greatest behavioral sensitivity differences between D/deaf and hearing participants (9, 11, 12). Although the boundaries of subregions were identified on flattened representations, depicting an inherently curved structure on a two-dimensional surface necessarily introduces some distortion, and this can vary across individuals depending on the position and curvature around the central point (*SI Appendix*, Fig. S2). Therefore, the actual boundaries were backtransformed to the surface defined on the high-resolution volume, and all structural metrics (cortical volume, thickness, and surface area) were derived from the original three-dimensional cortical manifold, where distances on the curved surface are preserved and not subject to distortions introduced by the flattening process. Statistical details are summarized in *SI Appendix*, Table S2.

#### V1 cortical volume.

**Fig. 4.**
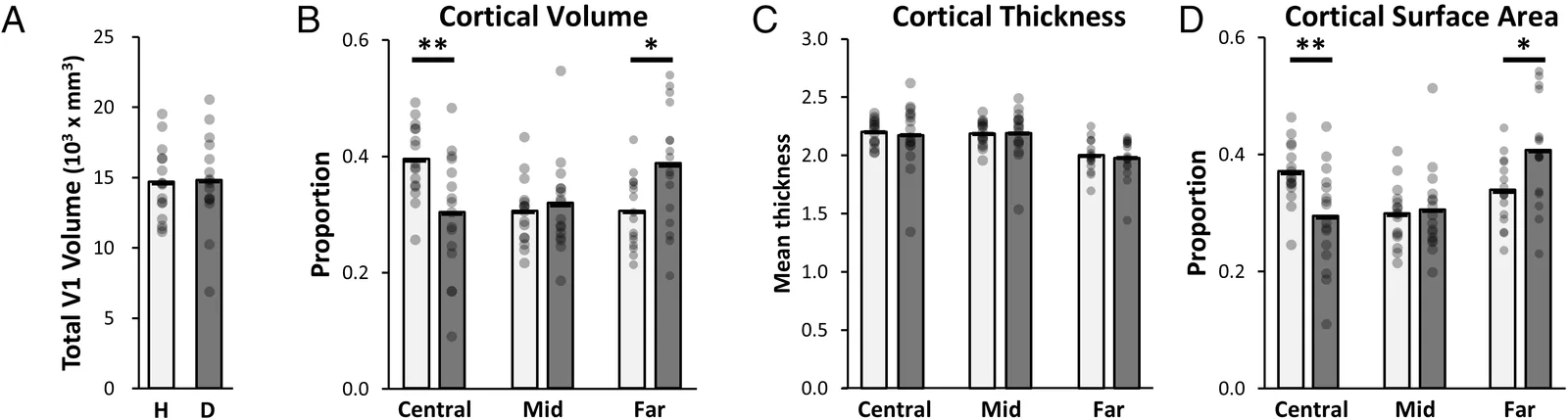
Effects of deafness on visual field representations in V1. Dots represent individual participant data and bars represent the mean for each group. (*A*) Total V1 volume (sum of left and right) for Hearing (H, light gray, N = 16) and D/deaf (D, dark gray, N = 16) groups. (*B*) Mean proportion of V1 cortical volume in subregions representing the Central (0 to 15°), Mid-peripheral (Mid, 15 to 39°), and Far-peripheral (Far, 39 to 72°) visual field. (*C*) Mean cortical thickness. (*D*) Mean cortical surface area proportions. **P < 0.01, *P < 0.05.

First, we compared the total cortical volume of V1. We found no difference between D/deaf and hearing groups (Fig. 4*A*) (t(30)= 0.154, *P* = 0.878, d = 0.055, 95% CI [−0.639, 0.747]), in agreement with a previous study that compared overall size of V1 between D/deaf and hearing adults within a smaller, more central visual field representation (±30°) (25). Next, we compared the relative volume of the three visual field representations in V1 between D/deaf and hearing groups, using proportional volume to correct for individual differences in overall V1 size (Fig. 4*B*). A mixed factorial ANOVA revealed a significant interaction between subregion and group (F(2,60) = 6.086, *P* = 0.004, ω^2^ = 0.141), indicating that volume is distributed differently across the three visual field representations between D/deaf and hearing groups. Between-group comparisons indicated a significantly larger central representation in hearing participants (t(30) = −2.984, *P* = 0.018, d = −1.055, 95% CI [−1.789, −0.305]), no significant difference in the mid-peripheral representation (t(30) = 0.452, *P* = 0.655, d = 0.160, 95% CI [−0.536, 0.853]), and a significantly larger far-peripheral representation in D/deaf participants (t(30) = 2.613, *P* = 0.028, d = 0.924, 95% CI [0.186, 1.648]).

#### V1 cortical thickness.

As differences in cortical volume can be affected either by changes in cortical thickness or surface area (or both), we compared both measures separately between groups. We found no difference in overall mean cortical thickness of V1 between D/deaf and hearing groups (ANOVA; Group, F(1,30) = 0.062, *P* = 0.805, ω^2^ < 0.001; see Fig. 4*C*). There was a significant difference in thickness between eccentricity representations across both groups (subregion, F(1.502,45.070) = 47.466, *P* < 0.001, ω^2^ = 0.203, Greenhouse–Geisser corrected), reflecting well-known thickness differences in all human brains between the occipital pole, which represents the central visual field, and the calcarine sulcus, representing the periphery. Importantly, however, there was no significant interaction between region and group, suggesting cortical thickness does not account for the group differences in volume found above (subregion*group: F(1.502,45.070) = 0.204, *P* = 0.753, ω^2^ < 0.001).

#### V1 cortical surface area.

Cortical surface area of each eccentricity representation was compared next, again using relative proportions to correct for individual differences in overall V1 surface area (Fig. 4*D*). An ANOVA revealed a significant interaction between subregion and group (F(2,60) = 5.663, *P* = 0.006, ω^2^ = 0.131), as seen in the volume measure. Between-group comparisons indicated a significantly larger central representation in hearing participants (t(30) = −2.978, *P* = 0.018, d = −1.053, 95% CI [−1.787, −0.303]), no significant difference in the mid-peripheral representation (t(30) = 0.275, *P* = 0.786, d = 0.097, 95% CI [−0.597, 0.790]), and a significantly larger far-peripheral representation in D/deaf participants (t(30) = 2.540, *P* = 0.034, d= 0.898, CI [0.162, 1.620]). This suggests that volumetric differences between groups reflect changes in surface area rather than cortical thickness.

### Effects of Visual Language Use.

**Fig. 5.**
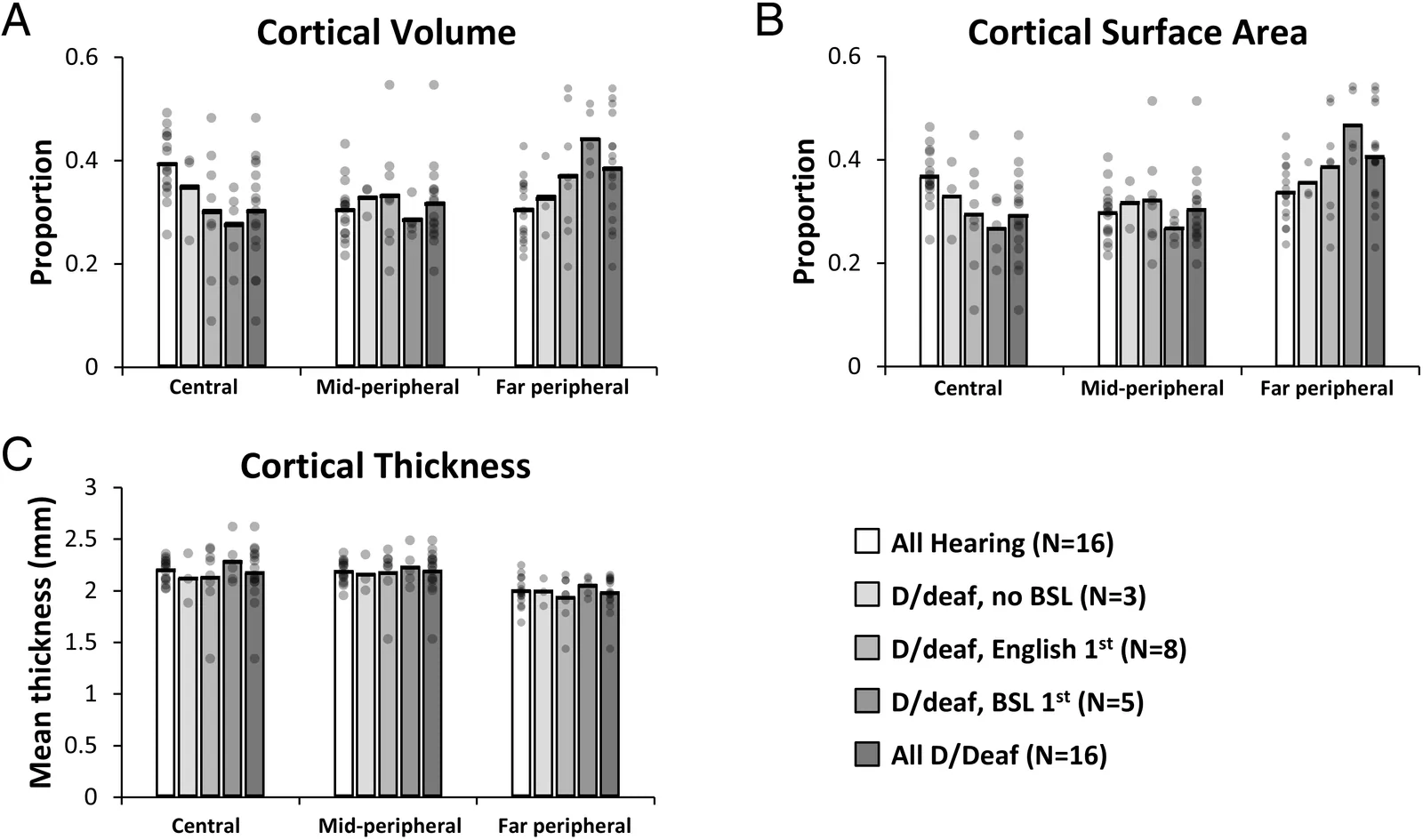
Effects of language on visual field representations in V1. (*A*) Volume proportions; (*B*) Surface area proportions; (*C*) Mean cortical thickness; Central region = 0 to 15°; Mid-peripheral region = 15 to 39°; Far-peripheral region = 39 to 72°. Legend applies to all panels. All Hearing: Hearing participants with English as a first language and no BSL; D/deaf, no BSL: Deaf participants with no BSL experience; D/deaf, English 1st: Deaf participants with English as a first language; D/deaf, BSL 1st: Deaf participants with BSL as a first language; All D/deaf: All deaf participants combined.

As most of our D/deaf participants were frequent users of British Sign Language (BSL) for communication, we also performed an exploratory comparison to evaluate our results in the context of sign language experience. Based on questionnaire data (tabulated in Table 1), D/deaf participants were divided into three groups: those whose first language was BSL (N = 5), those whose first language was English but who later learned and routinely use BSL (N = 8) and those without sign language experience (N = 3). Structural measurements within the three V1 subregions were compared between the different D/deaf subgroups and hearing controls (Fig. 5). The comparison reveals that the peripheral representations are largest (and central representations smallest) in the group who learned BSL as a first language, followed by BSL users with English as a first language and then nonsigning D/deaf individuals. Although sample sizes are too small to compute valid statistics, there is a clear trend suggesting that use of a visual sign language contributes to the central/peripheral trade-off of visual field representations in V1.

## Discussion

We mapped out the far-peripheral visual representations in early visual structures of D/deaf and hearing adults. We found compensatory plasticity in the LGN and V1 as a consequence of atypical lifelong sensory experience. We reveal a redistribution of neural resources in early D/deaf individuals, with a larger cortical surface representation of the periphery, at a cost of smaller representations of the central visual field. This central/peripheral trade-off in neural resources may explain behavioral differences reported between D/deaf and hearing individuals where numerous behavioral studies demonstrate superior visual sensitivity in early D/deaf individuals (1, 2, 12, 46), particularly to far-peripheral stimuli (9–11, 47).

Some recent research has investigated whether subcortical changes are present in response in early D/deaf individuals. While we found no significant difference in overall LGN volume in D/deaf participants, one study reported significantly smaller volumes in several subcortical nuclei, including the left LGN, compared to hearing participants (48) (*SI Appendix*). Studies evaluating differences in the early visual cortex of D/deaf individuals compared to controls have reported variable findings. One study found that cortical volume in a region around the calcarine sulcus (representing primary visual cortex) was larger in deaf than hearing participants (49), in contrast to our study, which found no difference in total V1 volume between groups. However, the calcarine region in that study was traced anatomically in individual participants guided by Brodmann’s area 17 and included the cortex beyond the parieto-occipital sulcus, which the authors acknowledge may imperfectly capture the actual boundaries of striate cortex (V1). Studies in which V1 was defined using functional, retinotopically defined boundaries limited to 30 to 37.5° eccentricity found either no difference (25) or thinner visual cortex and larger population receptive field sizes (34). The current study expanded the visual field representation measured and covered the far periphery where differences in visual sensitivity are most pronounced and potentially beneficial, beyond 37.5° (9, 11). In doing so, we found a peripheral shift in the proportion of active voxels in the D/deaf group. This may reflect underlying changes in both structure and function (as attention can also drive the number of voxels above threshold), suggesting that both types of changes may support improved visual performance in the near periphery (<30°). However, the increased far-peripheral surface area representation in V1 suggests that structural changes may be the main driving factor improving visual performance beyond 39°.

### The Role of Visual Sign Language Experience.

Most of our D/deaf participants were frequent users of BSL for communication, leading to the possibility that visual experience through signing might contribute to the neural changes reported here. Although we observed the same pattern of central/peripheral differences between D/deaf and hearing groups in both hemispheres, they were most pronounced in the left hemisphere (*SI Appendix*, Fig. S3). A left hemisphere bias in D/deaf individuals has also been reported in visual motion brain regions (50) and may underpin the right visual field (left hemisphere) advantage reported in D/deaf and hearing signers compared to hearing nonsigners (3, 9, 15). Although our study was not designed to examine the effects of sign language use explicitly, we took advantage of the fact that our D/deaf participants differed in language experience (Table 1) to explore the possible role of visual signing. We found a compelling trend suggesting that deafness drives the structural changes in V1, with sign language exposure having an additive effect (Fig. 5). This difference in sign language use across D/deaf participants may in part explain greater variability in structural measurements in the D/deaf group.

Although sign language gestures are found to primarily occur within 3 to 12° eccentricity in the lower visual field (51), the signing speed and large visual spatiotemporal area utilized in signed language may improve visual detection beyond this region (6, 52). We found the largest differences in primary visual cortex between D/deaf and hearing groups at eccentricities well beyond this range. A prior study using voxel-based morphometry in D/deaf individuals found that gray matter concentration in the anterior part of V1/V2 (representing peripheral vision) was negatively correlated with the age at which they learned sign language (53). This supports our finding that participants who learned BSL as their first language showed the largest increase in the far-peripheral representation of V1. An fMRI study that reported greater activity in primary auditory cortex in D/deaf native signers for near peripheral (11 to 15°) compared to perifoveal stimuli suggests that use of sign language in this part of the visual field may be supported in part by other brain regions (20).

Numerous studies using hearing signers as a control group have demonstrated that although sign language experience does confer visual performance advantages, these are well below the level of D/deaf signers (1, 6, 14, 15, 18, 47, 52). This is particularly true in far-peripheral (>30°) tasks both in humans (9), and in cats, who do not benefit from sign language experience (10). Moreover, although Allen and colleagues found a larger volume in the calcarine sulcus of D/deaf, signing individuals, there were no significant differences between hearing signers and hearing nonsigners (49). A recent study evaluating functional connectivity from auditory cortex to other brain structures provides further evidence that lack of auditory input is the main driver for differences between D/deaf and hearing groups, while the length of sign language experience accounts for additional individual differences to a lesser extent (54). Therefore, it is likely that deafness, even without sign language experience, influences visual pathways and behavior. Nevertheless, our exploratory analysis provides interesting pilot data to support the need for further research into the role of visual sign language experience in modifying near- and far-peripheral visual field representations.

### Methodological Considerations.

#### Apparent shift of eccentricity distributions and “wraparound effect”.

Eccentricity histograms displayed in Figs. 1*C* and 2 *E*–*G* represent the relative proportion of functionally active voxels within a region, effectively “weighted” by signal strength (i.e., the number of voxels above coherence threshold). This results in an apparent shift in the peak of the distributions and reduction in foveal responses, which is a product of three main factors. First, our wide-field expanding rings must traverse a larger portion of the visual field (0 to 72°) within roughly the same period as used in conventional retinotopic mapping (usually 0 to 15°), or fine foveal mapping (0 to 5°) (55), resulting in coarser sampling. Second, phase-encoded retinotopic stimuli are by nature continuous and cyclical, such that the stimulus “wraps around” at the end of the cycle when both fovea and periphery are stimulated, which also reduces the precision and resolution of mapping of the fovea. Third, a single voxel may contain neurons representing a range of eccentricities, which will shift the central tendency toward mid-eccentricities, i.e., the partial volume effect. An example of this effect can be observed in a study using a similar metric in the LGN [(56), figure 7B). By comparison, our structural measurements (volume, surface area, and thickness) were based on continuous, unthresholded maps that included all voxels within each region to avoid any functional biases and undersampling (Figs. 3 and 4). The wraparound effect is not an issue for the three hand-drawn subregions of interest used for structural measures, as voxels with similar phases representing the fovea and far periphery are anatomically distant from one another. Crucially, however, all methods and analyses were consistent across both hearing and D/deaf groups, making it unlikely that any systematic biases could explain group differences.

#### Defining V1 regions of interest.

We chose to use retinotopy data as the most accurate representation of the boundaries of primary visual cortex in each individual, particularly in the D/deaf population for whom an atlas does not exist. In phase-encoded mapping, boundaries between areas are indicated by a reversal in the phase of the response to rotating flickering checkerboard wedges at the upper and lower vertical meridians. As the vertical extent of the stimulus was limited in the scanner bore, borders were drawn by hand guided by phase reversals where possible and extrapolated using curvature information indicating the edge of the calcarine sulcus out to the horizontal extent of the activity (*Methods* and Fig. 2 *A*–*C* and *SI Appendix*, Fig. S2). Previous research has established that the boundaries of human V1 can be predicted by cortical anatomy, consistently following the banks of the calcarine sulcus across individuals (43, 57, 58). Boundaries were checked by three different observers (A.T.L., K.Y., and H.A.B.), including one naive to whether participants were D/deaf or hearing (K.Y.).

As a second, more “objective” measure to circumvent any potential biases, V1 definitions from two separate validated atlases were also transformed into each participant’s left and right occipital lobes in the T1-weighted structural volume. Qualitative comparisons revealed that V1 boundaries defined in the current study in both deaf and hearing groups matched closely to those in both atlases, suggesting that V1 borders do not develop differently in D/deaf individuals. Future research using finer sampling and higher-resolution retinotopic mapping methods could be used to provide more precise quantitative comparisons. Nevertheless, the multiple methods used to delineate V1 in the current study were applied equally to all participant data, and most importantly, there was no evidence of systematic bias boundaries between hearing and D/deaf groups. The fact that two independent atlas-based V1 representations produced nearly identical eccentricity distributions and resulted in the same group differences observed using retinotopically derived V1 also argues strongly against potential biases (Fig. 2 *E*–*G*).

The use of phase-encoded (“traveling wave”) stimuli to map eccentricity for such a large portion of the visual field (0 to 72°) may have limited accuracy, particularly in capturing the inner and outer boundaries of V1. Experiments comparing stimulus methods employing different spatial and temporal parameters have documented variations and biases in retinotopic map estimates, particularly at the representation of stimulus edges (59–61). We prioritized the superior signal-to-noise ratio offered by traveling wave stimuli, with the assumption that any mapping inaccuracies would apply equally to both hearing and deaf groups. However, as mentioned above, the coarser resolution afforded by wide-field retinotopic mapping may have reduced our estimates of the foveal representation, which normally requires higher-resolution stimuli to measure using fMRI (55), resulting in an underestimate of the central subregion of V1. A second concern is the possible overestimation of the subregion representing the far periphery due to the presence of visually responsive extrastriate areas with known peripheral biases immediately anterior to V1, such as area prostriata (62) and retrosplenial cortex (63). Indeed, evidence of both of these effects can be seen in the single participant shown in Fig. 2*C* when comparing atlas-based and retinotopic-based V1 boundaries. Group data shown in Fig. 4*D* estimate that on average, the central 0 to 15° representation comprises roughly one third of the total V1 area measured (Fig. 4*D*), while converging anatomical, fMRI and neurophysiological mapping methods suggest this proportion should be closer to one half (60, 64–67). Furthermore, our estimate of the proportion of V1 representing the visual field beyond 39° is larger than that based on human anatomical data and macaque measurements (64, 65, 67). Again, however, any inaccuracies introduced by the stimulus methods would apply to both hearing and D/deaf groups.

### Potential Neural Mechanisms.

Differences in neural resources within the LGN and V1 may partly reflect upstream differences in projections from the retina. Codina and colleagues reported that the retinal nerve fiber layer containing ganglion cell axons from peripheral retina was thicker in D/deaf participants, while the region containing central projections was thicker in hearing participants (12). These differences could in turn lead to changes in downstream projection areas. Our measurements in the LGN suggest that the redistribution of cortical representations is at least in part mediated by feedforward connections. Animal studies in the somatosensory system indicate that thalamic projections can influence cortical plasticity further along the processing pathway (68). Moreover, differences in the visual field distribution of electrophysiological responses from the retina and cortex in D/deaf and hearing groups suggest that additional modifications may occur between the retina and V1 (69). The current study using functional MRI supports the idea that visual experience can shape the distribution of resources devoted to central and peripheral visual processing and that this may originate precortically. One possibility is that differences in primary visual cortex in D/deaf individuals are entirely inherited from the LGN. Previous research suggests that connections between the LGN and V1 may be relatively stable (70, 71). Even in early blindness, where cross-modal plasticity appears to strengthen cortico-cortical connections between primary visual and auditory cortices, thalamocortical connections between V1 and the LGN remain unchanged (33). Interestingly, a recent study found evidence of plasticity in D/deaf individuals in subcortical-to-cortical connectivity to auditory cortex, but not to visual cortex, offering further support for stable connections within the visual pathway (72). Whether additional reorganization occurs within V1 or between V1 and other cortical areas remains to be seen.

What mechanisms might mediate neural changes in the early visual system? Evidence suggests that typical neural development is characterized by substantial loss of neurons and synapses and that neuronal activity specifies selective elimination and maintenance of cortical connections (73, 74). Therefore, visual functions most likely to adapt in response to early onset deafness are those which would under normal development benefit from the convergence of both auditory and visual stimuli such as peripheral vision (1). Given the well-documented crossmodal recruitment of auditory cortex in D/deaf individuals, one possibility is that changes in the peripheral representation in V1 might reflect increased input via direct connections from auditory and superior temporal regions to calcarine cortex (75, 76). Another important factor to consider is that some of the deaf participants included were known to be genetically D/deaf, and some had unknown causes behind their deafness, which could include an unidentified genetic precursor. Future studies would be beneficial in investigating possible links between genetic deafness and visual enhancements and cortical plasticity.

## Conclusion

Until now, remapping in primary visual cortex has only been found when the visual system itself is compromised by disease or dysfunction (27, 28). Our current finding indicates that the sculpting of early visual maps during development can be driven by the demands that are placed on vision due to lifelong deafness, even in the absence of any visual deficit. The developmental plasticity of the brain and specifically the LGN and V1 therefore allows it to be tuned by, and probably optimized to, visual behavior.

## Materials and Methods

### Participants.

The study included 32 participants: 16 early D/deaf individuals (mean age = 34.13, range = 20 to 48 y, 5 females) and 16 hearing individuals (mean age = 29.61, range = 20 to 48 y, 5 females) naive to any sign language, including BSL. There was no significant difference in age between the groups (t(30) = 1.291, *P* = 0.207). All participants were recruited through advertisements at the University of York, and additional D/deaf participants were invited to take part by collaborators, Prof. Charlotte Codina and Dr. David Buckley from the University of Sheffield. D/deaf participants were also paid for participating and reimbursed for their travel expenses, if traveling from outside of York. All participants gave informed consent in accordance with the Declaration of Helsinki. A BSL interpreter was present throughout the sessions for BSL-using D/deaf participants to ensure they understood the instructions and purpose of the study and to answer any questions. Each D/deaf participant also filled out a brief questionnaire regarding the known etiology of deafness and their sign language experience (Table 1). All D/deaf participants reported severe to profound hearing loss in both ears (>70 db) from early childhood (<4 y). One participant became deaf as a result of rubella and two as a result of in utero measles, either of which could have caused a visually associated impairment. These participants underwent baseline visual acuity, pupillary reactions, and fundus examinations, and on careful ophthalmic investigations, no abnormalities were found. All participants had good visual acuity in either eye with a minimum best corrected vision of 0.20 logMAR units (ETDRS chart), and none had nystagmus or any neurological conditions affecting vision or the vestibular system (e.g., Waardenburg or Usher syndrome). The majority (10/16) of D/deaf participants also underwent more extensive vision testing by a qualified orthoptist (Codina) as part of other studies at the University of Sheffield. This included comprehensive ocular motility testing of horizontal and vertical saccades, vestibular ocular reflex and smooth pursuits to all positions of gaze, with no abnormalities found. The study was approved by the York Neuroimaging Centre Research Governance Committee.

### Experimental Design.

Participants viewed the stimuli at a distance of 275 mm from a supine position through a wide mirror mounted on the head coil, allowing them to see the projection on a custom in-bore acrylic screen (3,050 mm × 2,030 mm) mounted behind the head coil. The head of each participant was stabilized with foam pads placed inside the head coil and a forehead Velcro-strap to reduce motion artifacts. To increase the restricted field of view normally imposed by the MRI scanner, each hemifield was tested in separate functional scans allowing the stimulus to extend to 72° along the horizontal meridian. Participants viewed stimuli passively, continuously fixating a small cross placed either on the left or right side of the screen depending on which hemifield was being tested. After the first pilot sessions (4 hearing, 5 D/deaf), the head was tilted slightly (ca. 3°) toward the fixation cross to maximize participant comfort, field of view, and fixation and head stability. Instructions were communicated in the MRI scanner between scans, verbally to hearing participants and visually via PowerPoint slides to D/deaf participants. The D/deaf participants were asked to push down a button when a message appeared on the screen and release it when they had read the message and were happy to proceed, and hearing participants responded verbally. Every participant was provided with an emergency buzzer, which could be pressed at any time if they wished to quit the scanning procedure.

### Retinotopic Mapping.

To identify visual maps and target the far periphery, this study used a wide-field retinotopic approach to map visual field eccentricity to reaching well beyond conventional mapping studies, which typically do not extend further than ca. 20° (35–38, 77). This method was used to identify primary visual cortex (V1) and map visual field eccentricity out to 72° (Figs. 1*A* and 2 *A* and *B*), including a region corresponding to the far periphery (>39°) (Fig. 3*E*) where previous studies found the greatest behavioral sensitivity differences between D/deaf and hearing participants (9). The subregions of interest were selected to contain roughly the same number of voxels. Two versions of the retinotopic stimulus paradigm were used, due to a change in stimulus display equipment in the York Neuroimaging Centre. The first version was used during pilot scanning of five D/deaf and four hearing participants. The remaining D/deaf participants and hearing controls were scanned with a second version whose parameters closely matched that of the first. Both versions included wedge and ring stimuli, both containing high contrast reversing checker boards (100% luminance contrast, mean luminance = 98 cd/m^2^). The order in which the hemifield stimuli were presented was counterbalanced across all participants.

The first version of the retinotopic stimuli was generated with Matlab (version R2012a; The MathWorks, Natick, MA) and presented with MatVis (Neurometrics Institute) and rear-projected to participants through the scanner bore with a Dukane 8942 ImagePro (Dukane Corporation, St. Charles, IL, USA). The mean luminance of the display was 97.87 cd/m^2^ measured with a Minolta Luminance Meter (LS −100/LS 110). The order in which the hemifield stimuli were presented was counterbalanced across all participants. The rotating wedge stimulus comprised 30° of a full circle and extended horizontally to 72° visual angle in each hemifield and vertically ca. ±20° visual angle from fixation (Fig. 2*B*). The wedge stimulus reversed in contrast at 6 Hz and stepped in 11.25° polar angle increments beginning at the upper vertical meridian within both hemifield conditions. This stimulus was used for participants H1, H13, H14, H15, H16, D11, D12, D13, D14, and D15.

The second version of stimuli was generated with Psykinematix 1.4 (Beaudot, 2009) on a Mac Mini OS X (Apple Inc., Cupertino, CA) and projected to participants through the scanner bore with a PROPixx DLP LED projector (VPixx Technologies, Saint-Bruno, QC, Canada). The mean luminance of the display was 98.6 cd/m^2^ (min–max luminance 2.42 to 199.7 cd/m^2^), measured with a Spyder 3 Pro calibration device (Datacolor, Lawrenceville, NJ). The wedge stimulus contrast reversed at a rate of 4 Hz and stepped in 15° increments and beginning at the upper vertical meridian for the right hemifield, and at the lower vertical meridian in the left hemifield. The expanding annulus in both stimulus sets had a total annular width of 14° and expanded in increments of 4.65° (one check width). As the rings approached the edge of the stimulated field, each ring was replaced by a new one originating from the center of fixation. A fixation cross, a gray “+” sign, 0.87° in size or a red + sign, 0.6° in size, was present throughout the entire scan. This stimulus was used for participants H2, H3, H4, H5, H6, H7, H8, H9, H10, H11, H12, D1, D2, D3, D4, D5, D6, D7, D8, D9, D10, and D16.

### MRI Acquisition.

MRI data were acquired using a 16-channel posterior brain array coil (Nova Medical, Wilmington, MA) in a GE 3 Tesla Signa Excite HD scanner (GE Healthcare, Chicago, IL) at the York Neuroimaging Centre. High-resolution structural data of the entire brain were acquired with high-resolution T1-weighted isotropic scans, (TR, 8 ms; TE, 3 ms; flip angle, 12°; matrix size, 256 × 256; FOV, 256 mm; 176 slices; slice thickness, 1 mm; voxel size, 1 × 1 × 1 mm^3^). Structural proton density scans (TR, 2.7 s; TE, 36 ms; flip angle, 90°; matrix size, 512 × 512; FOV, 192 mm; 39 slices; slice thickness, 2 mm; voxel size, 0.37 × 0.37 mm^3^) were also acquired prior to and in the same plane as each functional session in order to aid the coregistration of functional data with structural volumes, and identify the LGN. Functional data were acquired with a BOLD T2* EPI sequence (TR, 3 s; TE, 30 ms; flip angle 90°; matrix size, 128 × 128; FOV, 192 mm; 39 slices; slice thickness, 2 mm; voxel size, 1.5 × 1.5 × 2 mm^3^). Each participant underwent one high-resolution structural scan, and two sessions consisting of a structural proton density scan followed by functional runs mapping the visual field (one expanding ring, one rotating wedge), testing each hemifield separately.

### MRI Data Preprocessing.

T1-weighted images were corrected for magnetic spatial inhomogeneities with FMRIB’s Automated Segmentation Tool (FAST) (45). A T2* gradient echo scan was acquired in a subset of participants (3 hearing, 4 D/deaf), which was used to aid further in inhomogeneity correction. These data were then processed with the FreeSurfer 6.0 analysis suite to segment and reconstruct the gray matter surface (78). The occipital lobe of the reconstructed image was then corrected by manually segmenting and topology checking in ITK-Snap (79). These segmentations were used to create flattened cortical representations (80) on which the retinotopic data were displayed using the mrVista toolbox written in Matlab (https://web.stanford.edu/group/vista/cgi-bin/wiki/index.php/MrVista) (38).

Phase-encoded retinotopic scans were processed with the mrVista toolbox, run in Matlab. In order to correct for motion, the T2* functional volumes were aligned to the first acquired volume of the session. Data were slice time corrected and high-pass filtered to remove baseline drifts. MrVista corrects for motion within and between functional volumes and uses a mutual information motion correction algorithm (81). The corrected functional scans were coregistered to the coordinate space of the high-resolution structural image for each participant using FMRIB’s Linear Image Registration Tool (FLIRT) (82) and the Nestares alignment code (81), which is part of the mrVista toolbox.

### Verification of Fixation.

Wide-field retinotopic mapping was enabled by reducing the distance to the screen and tilting the head slightly (ca. 3°) to stimulate one hemifield at a time. This configuration prevented the mounting of an eye tracking device, and therefore fixation could not be monitored directly. Fortunately, the majority of our D/deaf participants had taken part in vision experiments before and were experienced with maintaining fixation. However, to check for fixation stability, we reasoned that if participants were fixating eccentrically away from the fixation mark toward the stimulus, part of the stimulus should cross-over the vertical midline resulting in increased activity in the ipsilateral hemisphere, particularly in the central representation. The total number of stimulus-driven voxels responding above a coherence threshold of 0.23 (*P* < 0.01, uncorrected) was calculated for each participant in each hemisphere for each hemifield stimulus condition, and data were combined (summed) for both ipsilateral hemispheres and both contralateral hemispheres. A ratio of ipsilateral:contralateral activity was also calculated for each participant. We found no significant difference between D/deaf and hearing groups in either the total number of ipsilateral V1 voxels activated (t(30) = 0.912, *P* = 0.369) or in the ratio of ipsilateral:contralateral activity (log(ratio) taken for statistics; t(30) = 0.047, *P* = 0.963). The individual data, means, and ratios of ipsilateral:contralateral V1 activity are shown in *SI Appendix*, Table S1.

### Defining Regions of Interest (ROIs).

#### LGN.

The left and right lateral geniculate nuclei were identified in each participant using high-resolution proton density scans taken in the same slice orientation and location as functional scans (*MRI Protocol*) for optimum visualization of subcortical structures (Fig. 1*B*) (39, 41, 83). Regions of interest were identified on anonymized scans (K.Y.) and checked by a second individual (H.A.B.) such that the identifier was unaware of whether scans belonged to D/deaf or hearing participants. The LGN could not be identified clearly in one D/deaf and one hearing participant, and hence LGN analyses included 15 participants per group. The accuracy of LGN selection and coregistration was assessed by comparing it with the position of the LGN and another nearly visually responsive subcortical structure, the pulvinar, based on a standardized atlas, Thalamus Optimized Multi Atlas Segmentation (THOMAS) (40). First, the T1-weighted images for each participant were coregistered with the MNI152 standard brain using the linear coregistration tool, FLIRT in the FSL toolbox (FMRIB Software Library) (45). Next, the cumulative sum of all LGN regions across all participants was computed within MNI space. Finally, the cumulative LGN definition across all participants was displayed along with the LGN and the pulvinar from the THOMAS atlas (*SI Appendix*, Fig. S1).

#### Retinotopically guided V1 definitions.

The delineation of the boundaries of primary visual cortex, V1, was based on previous literature, establishing the identifying features of visual areas based on phase-encoded data (35–38). To aid data visualization, the phase-encoded retinotopic data were displayed on a flattened representation of the occipital cortex in order to identify boundaries of primary visual cortex (V1) (80). Curvature was also encoded on the flattened surface using a gray scale, in which darker regions denoted sulci and lighter regions gyri. As the functional definition of the vertical meridian was limited by the vertical extent of our display in the scanner, we used additional anatomical landmarks, particularly the calcarine sulcus, to define the entire extent of V1 which follows the shape/anatomical definition of this sulcus (*SI Appendix*, Fig. S2). V1 boundaries were checked and verified by three different experimenters (A.T.L., K.Y., and H.A.B.), including one (K.Y.) who was naïve to the group identity of participants (D/deaf or hearing). As the flattening process distorts the distance and area measurements within the 2D dimensions, all coordinates were transformed into the 3D cortical manifold and measurements extracted thereafter (36).

#### Atlas-based V1 definitions.

In addition to the methods described above to define the outer boundaries of V1 based on individual retinotopy data, two other versions of V1 boundaries were generated by applying two different publicly available atlases—the Benson atlas (43) and the Human Connectome Project HCP _ MMP1.0 parcellation (44) to the cortical surface. This allowed us to compare group differences throughout V1 using an independent, surface-based method of boundary estimation based primarily on anatomical features while avoiding boundary uncertainties where functional retinotopic data were missing or noisy.

To generate V1 regions of interest using the Benson atlas, the cortical surface for each participant was segmented from high-resolution T1-weighted structural data using FreeSurfer 6.0, and aligned to the fsaverage, the FreeSurfer average brain (78). Cortical segmentation was successful in all 16 participants in each group. The “neuropythy” toolbox function “benson14_retinotopy” was then used to estimate the Benson atlas-defined boundaries of V1 on the individual FreeSurfer surface ribbon of each participant (43). The Benson atlas estimates the location and retinotopic organization of V1 from 0 to 90° of eccentricity based on anatomical features and has been functionally validated for 0 to 20° eccentricity (https://nben.net/Retinotopy-Tutorial/#atlas-benson2014). This surface-based V1 label was then converted back into volumetric space (FreeSurfer’s .mgz format) and subsequently converted to NIfTI (.nii.gz) format. The V1 labels for the left and right hemispheres were then combined and binarized to create a single V1 ROI mask. All regions of interest were carefully checked and compared within the T1-weighted structural MRI volume for each participant in each hemisphere using the visualization tool FSLeyes (v.1.9.0; FMRIB Software Library) (45). The Benson definition of V1 did not align accurately in the right hemispheres of two hearing participants. Therefore, for these two participants, only the left hemispheres were used, resulting in accurate surface-based definitions of V1 in 62 out of 64 hemispheres. The V1 Benson ROI was then transformed into a format compatible with mrVista.

To generate V1 regions of interest using the Human Connectome Project HCP_MMP1.0 parcellation, each participant’s high-resolution T1-weighted structural scan was run-through the HCP pipeline, version 4.0.0 (https://www.humanconnectome.org) (84). The primary output volume utilized from this pipeline was the AC/PC aligned T1w image (T1w_acpc_dc_restore_brain.nii.gz). To define the V1 ROI, the HCP atlas labels were downloaded in surface format, specifically retrieving V1 labels for the left and right hemispheres. These atlas surface labels were then mapped to individual subjects’ native surface, constrained by the “ribbon” (the pial and white matter surfaces), with the resulting labels saved in volumetric space (.nii.gz) for each hemisphere separately. Next, the HCP output volume (T1w_acpc_dc_restore_brain.nii.gz) was realigned to each individual’s original T1w space using a linear registration tool, and the corresponding transformation matrix was saved. This transformation matrix was then applied to all Glasser atlas labels for both left and right hemispheres using nearest neighbor interpolation. From these transformed labels, the V1 left (V1L) and V1 right (V1R) ROIs were selected and binarized into separate volumetric (.nii.gz) masks for each participant. All regions of interest were carefully checked and compared within the T1-weighted structural MRI volume for each participant in each hemisphere using the visualization tool FSLeyes (v.1.9.0; FMRIB Software Library)(45). Data from one hearing and 3 D/deaf participants either failed to complete the HCP pipeline or yield an accurate transformation, resulting in 15 hearing and 12 D/deaf participants. The V1 ROIs were then transformed into a format compatible with mrVista.

### LGN and V1 Eccentricity Distributions.

Evaluating retinotopic maps within a small subcortical structure such as the LGN is problematic without high-resolution imaging, and hence we first performed an analysis based purely on functional data. The same procedure was also applied to the retinotopic and atlas definitions of V1 to allow for direct comparisons between the LGN and V1 regions. Functional voxels falling within each region of interest were analyzed from expanding ring scans (FFT analysis, phase yields preferred retinal eccentricity for each voxel). A coherence threshold of >0.23 (uncorrected *P* < 0.01) was applied to exclude noisy voxels. For each participant, phase data were converted into 72 binned eccentricities (1° each) for plotting and statistical analysis. For visualization purposes, these 72 bins were downsampled to 24 bins (3° each), while the 72-bin data were retained for statistical computations. For visualization, histograms representing the distribution of voxels as a function of eccentricity were generated for each participant and averaged across participants in each group (Figs. 1*C* and 2 *E*–*G*). For each participant, the cumulative number of active voxels within each bin representing 3° eccentricity was calculated across 24 bins, to include the entire extent of the stimulus, 0 to 72°. The proportion of total active voxels, across both hemispheres, was calculated within each bin for each participant. The proportions were then averaged across participants within each group (D/deaf, hearing), and SEM were also calculated. The LGN data (Fig. 1*C*) includes 15 participants per group. The V1 data include all 16 participants per group using retinotopically defined V1 boundaries (Fig. 2*E*) and boundaries defined by the Benson atlas (Fig. 2*F*) (43), but 15 hearing and 12 D/deaf participants using the Human Connectome Project atlas (Fig. 2*G*) (44). For statistical analysis, two-sample Kolmogorov–Smirnov tests were run to compare the 72-bin distributions of eccentricity representations between D/deaf and hearing groups for the LGN and the retinotopic and atlas definitions of V1. All computations and statistical analyses were performed in Matlab (version R2022a; The MathWorks, Natick, MA).

### V1 Subregion Definition and Measurement.

V1 was further subdivided into three roughly equal-sized subregions of interest, including a region corresponding to the far periphery (>39°) where previous studies found the greatest behavioral sensitivity differences between D/deaf and hearing participants (9, 11, 12). The regions of interest were selected to contain roughly the same number of voxels and represented three divisions of the visual field: central (0 to 15°); mid-peripheral (15 to 39°) and far-peripheral (39 to 72°), corresponding to central, mid-peripheral, and far-peripheral retinal locations (Fig. 3 and *SI Appendix*, Fig. S2). Eccentricity data were displayed on flattened cortex, restricted to the response phase window corresponding to the eccentricity ranges for each sub-ROI. This guided the manual selection of all cortical voxels within each subregion, not just those voxels which were active. Cortical volume (mm^3^), surface area (mm^2^), and gray matter thickness (mm) were extracted for each sub-ROI. The surface area measurements were made on the 3D cortical manifold, following the method used by Dougherty et al. (36). In this method, the visual areas are outlined on a 2D flat map, then transformed into the 3D manifold. The surface area was calculated by taking the coordinates belonging to the selected ROI and finding the nearest node on the 3D manifold describing the boundary of gray and white matter. The mean proportions reported were calculated by taking either the cortical volume or surface area sum of all sub-ROIs within V1 and dividing each sub-ROI by the total of each measure.

Cortical measurements were compared statistically between D/deaf and hearing groups in the entire cohort and also excluding the six D/deaf participants who did not undergo ocular motor and vestibular ocular reflex (VOR) testing to rule out any potential contribution of vestibular impairment (*SI Appendix*, Table S2).

## Supplementary Material

Appendix 01 (PDF)

Dataset S01 (XLSX)

## Data Availability

Anonymized spreadsheet data used to generate all graphs have been made available in the Supporting Information (*SI Appendix*, Dataset S1).
