# Supplementary material for: Retinotopic remapping of the visual system in deaf adults
Source: Proc Natl Acad Sci U S A. 2026 Sep 4;123(36):e2532413123. doi: 10.1073/pnas.2532413123 (PMC13552904; doi:10.1073/pnas.2532413123)
Supplement: Supplementary file 1 [file pnas.2532413123.sapp.pdf]

PNAS

Supporting Information for

Retinotopic remapping of the visual system in deaf adults

Alexandra T. Levine, Kate Yuen, André Gouws, Alex R. Wade, Antony B. Morland, Charlotte Codina, David Buckley, Heidi A. Baseler

Corresponding author: Heidi A. Baseler
Email: heidi.baseler@york.ac.uk

This PDF file includes:

Tables S1 to S2
Figures S1 to S3
Legends for Dataset S1
SI References

Other supporting materials for this manuscript include the following:

Dataset S1

Table S1. Fixation stability: V1 Ipsilateral vs. Contralateral Activity. The table represents the number of active (stimulus-driven) voxels, > 0.23 coherence ($p < 0.01$, uncorrected) in the ipsilateral and contralateral hemispheres in V1 defined retinotopically for each hearing (N=16) and each D/deaf (N=16) participant. Ipsi:Contra = Ratio of active voxels in the Ipsilateral:Contralateral hemispheres. For a full description see the main manuscript text under *Materials and Methods: Verification of fixation.*

	**V1 Ipsilateral**		**V1 Contralateral**		**Ratio (Ipsi:Contra)**	
	**Hearing**	**D/deaf**	**Hearing**	**D/deaf**	**Hearing**	**D/deaf**
	4876	4364	12577	11329	0.388	0.385
	4892	3356	10323	8730	0.474	0.384
	2356	2758	7843	10627	0.300	0.260
	6874	5479	14063	11407	0.489	0.480
	6303	5144	16924	11139	0.372	0.462
	7254	6420	12291	11754	0.590	0.546
	4539	4434	11917	12253	0.381	0.362
	5839	2474	9968	11487	0.586	0.215
	6408	5475	11188	11209	0.573	0.488
	4381	3440	9814	7918	0.446	0.434
	4042	5133	11759	11051	0.344	0.464
	5572	8386	11718	12735	0.476	0.659
	12104	5495	16844	12524	0.719	0.439
	4876	7236	12791	12746	0.381	0.568
	3210	5159	11622	6729	0.276	0.767
	5992	5004	10905	10571	0.549	0.473
Mean	**5594.88**	**4984.81**	**12034.19**	**10888.06**	**0.459**	**0.462**
SEM	**543.31**	**390.03**	**592.09**	**428.72**	**0.030**	**0.034**

Table S2. Cortical measurement statistics. Statistical comparisons of cortical measurements between D/deaf and hearing groups in the entire cohort (N = 32) and also when excluding the six D/deaf participants who did not undergo ocular motor and vestibular ocular reflex (VOR) testing to rule out any potential contribution of vestibular impairment (N = 26). D: D/deaf; H: Hearing. *Holm-Bonferroni corrected. **Greenhouse-Geisser corrected. Key statistical results remain, shown in bold.

	**All 32 participants (16 hearing, 16 D/deaf)**	**26 participants (16 hearing, 10 D/deaf)**
**Total V1 cortical volume**	t(30) = .154, p = .878, d = .055, 95% CI [-0.639, 0.747]	t(24) = .448, p = .658, d = .181, 95% CI [-0.613, 0.971]
**Volume (sub-region)**		
Group	F(1,30) = 3.076, p = .090, ω^2 = .032	F(1,24) = 6.298, p = .019, ω^2 = .096
ROI	F(2,60) = 0.056, p = .300, ω^2 = .007	F(2,48) = 0.946, p = .396, ω^2 < .001
Group X ROI	**F(2,60) = 6.086, p = .004, ω^2 = .141**	**F(2,48) = 3.418, p = .041, ω^2 = .088**
Posthoc t-tests:*		
Central D v H	t(30) = -2.984, p = .018, d = -1.055, 95% CI [-1.789, -0.305]	t(24) = -2.511, p = .057, d = -1.012, 95% CI [-1.843, -0.163]
Mid D v H	t(30) = 0.452, p = 0.655, d = .160, 95% CI [-0.536, 0.853]	t(24) = 0.478, p = 0.637, d = .193, 95% CI [-0.601, 0.983]
Far D v H	t(30) = 2.613, p = .028, d = .924, 95% CI [0.186, 1.648]	t(24) = 1.776, p = .176, d = .716, 95% CI [-0.107, 1.524]
**Thickness (sub-region)**		
Group	F(1,30) = .062, p = .805, ω^2 < .001	F(1,24) = 0.006, p = .940, ω^2 < .001
ROI **	F(1.502,45.070) = 47.466, p < .001, ω^2 = .203	F(1.411,33.864) = 42.753, p < .001, ω^2 = .209
Group X ROI **	F(1.502,45.070) = .204, p = .753, ω^2 < .001	F(1.411,33.864) = 0.621, p = .489, ω^2 < .001
**Surface area (sub-region)**		
Group	F(1,30) = 4.439, p = .044, ω^2 = .053	F(1,24) = 6.630, p = .017, ω^2 = .101
ROI	F(2,60) = 5.292, p = .008, ω^2 = .121	F(2,48) = 3.747, p = .031, ω^2 = .099
Group X ROI	**F(2,60) = 5.663, p = .006, ω^2 = .131**	**F(2,48) = 3.426, p = .041, ω^2 = .088**
Posthoc t-tests:*		
Central D v H	t(30) = -2.978, p = .018, d = -1.053, 95% CI [-1.787, -0.303]	t(24) = -2.597, p = .048, d = -1.047, 95% CI [-1.881, -0.194]
Mid D v H	t(30) = 0.275, p = .786, d = .097, 95% CI [-0.597, 0.790]	t(24) = 0.158, p = .876, d = .064, 95% CI [-0.727, 0.853]
Far D v H	t(30) = 2.540, p = .034, d = .898, 95% CI [0.162, 1.620]	t(24) = 1.873, p = .146, d= .755, 95% CI [-0.070, 1.566]

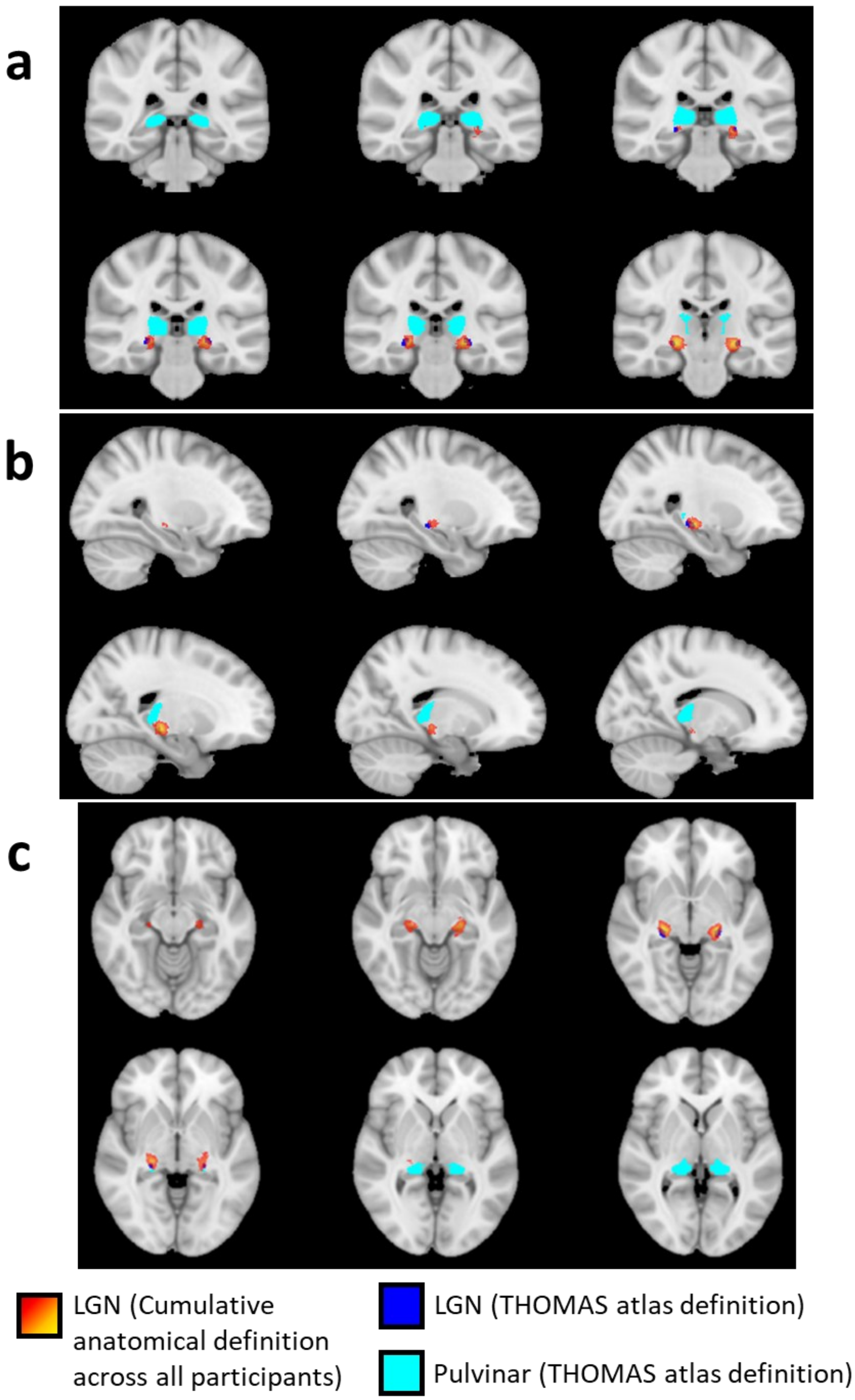

Fig. S1. Anatomical definition of the lateral geniculate nucleus (LGN) region of interest. (a-c) Views of the LGN and pulvinar displayed on the standard MNI brain (MNI152_T1_1mm_brain). All coordinates are provided in MNI152 space. Red/orange region represents the cumulative sum of all LGN regions anatomically defined in each participant, transformed to MNI standard space. Light blue region represents the pulvinar and dark blue, the LGN, as defined by the Thalamus Optimized Multi Atlas Segmentation (THOMAS) atlas **(1)**. (a) Coronal MNI slices ranging from y = 25 to 9; (b) Sagittal slices ranging from x = 27 to 16; (c) Axial slices ranging from z = -7 to 3.

Fig. S2 (a-h). Primary visual area (V1) and eccentricity sub-region boundaries, as defined in the study by retinotopic functional data, drawn on computationally flattened representations of the posterior occipital lobes. Each rectangular panel represents data for a single participant. (a-d): Hearing participants, H1-H16. (e-h): D/deaf participants, D1-D16. Left and right hemispheres shown on left and right for each participant, respectively. Each pair of images is oriented with the dorsal surface (Parieto-Occipital Sulcus, 'POS') towards the top of the image, ventral towards the bottom, posterior surface (Occipital Pole, 'OP') laterally and anterior (Calcarine Sulcus, 'CAL') medially. V1 boundaries and divisions between the three sub-regions are outlined in black. In each panel, the top pair of images shows the flattened surface without functional data. Gray scale represents anatomical surface curvature, with light gray denoting gyri and dark gray sulci. The middle panel shows images with superimposed rotating wedge data, and the bottom panel the expanding ring data (coherence threshold < 0.20). Color map legends for polar angle and eccentricity maps are shown to the left and right of each set of images and vary across participants depending on which of two mapping protocols was used. The first version of the retinotopic stimuli, in which the rotating wedge to map polar angle began at the upper vertical meridian in both hemifields, was used for participants H1, H13, H14, H15, H16, D11, D12, D13, D14 and D15. The second version of the retinotopic stimuli, in which the rotating wedge began at the upper vertical meridian for the right hemifield and at the lower vertical meridian in the left hemifield, was used for participants H2, H3, H4, H5, H6, H7, H8, H9, H10, H11, H12, D1, D2, D3, D4, D5, D6, D7, D8, D9, D10 and D16. See Methods, 'Retinotopic mapping', and Fig. 2a-d and Fig. 3 for further details. *H11: See Figure S2 (i).

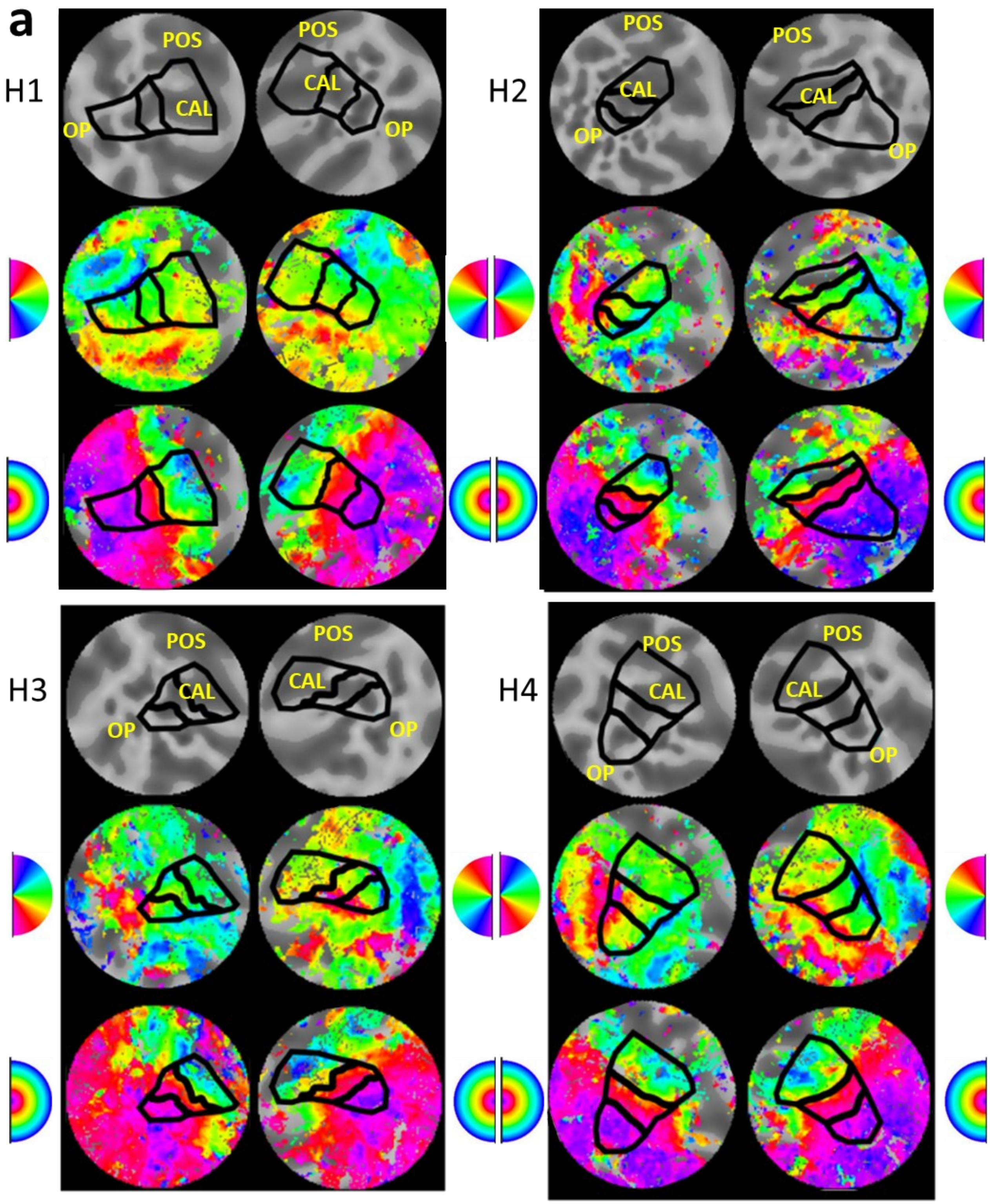
a
H1
POS
CAL
OP
POS
CAL
OP
H2
POS
CAL
OP
POS
CAL
OP
H3
POS
CAL
OP
POS
CAL
OP
H4
POS
CAL
OP
POS
CAL
OP

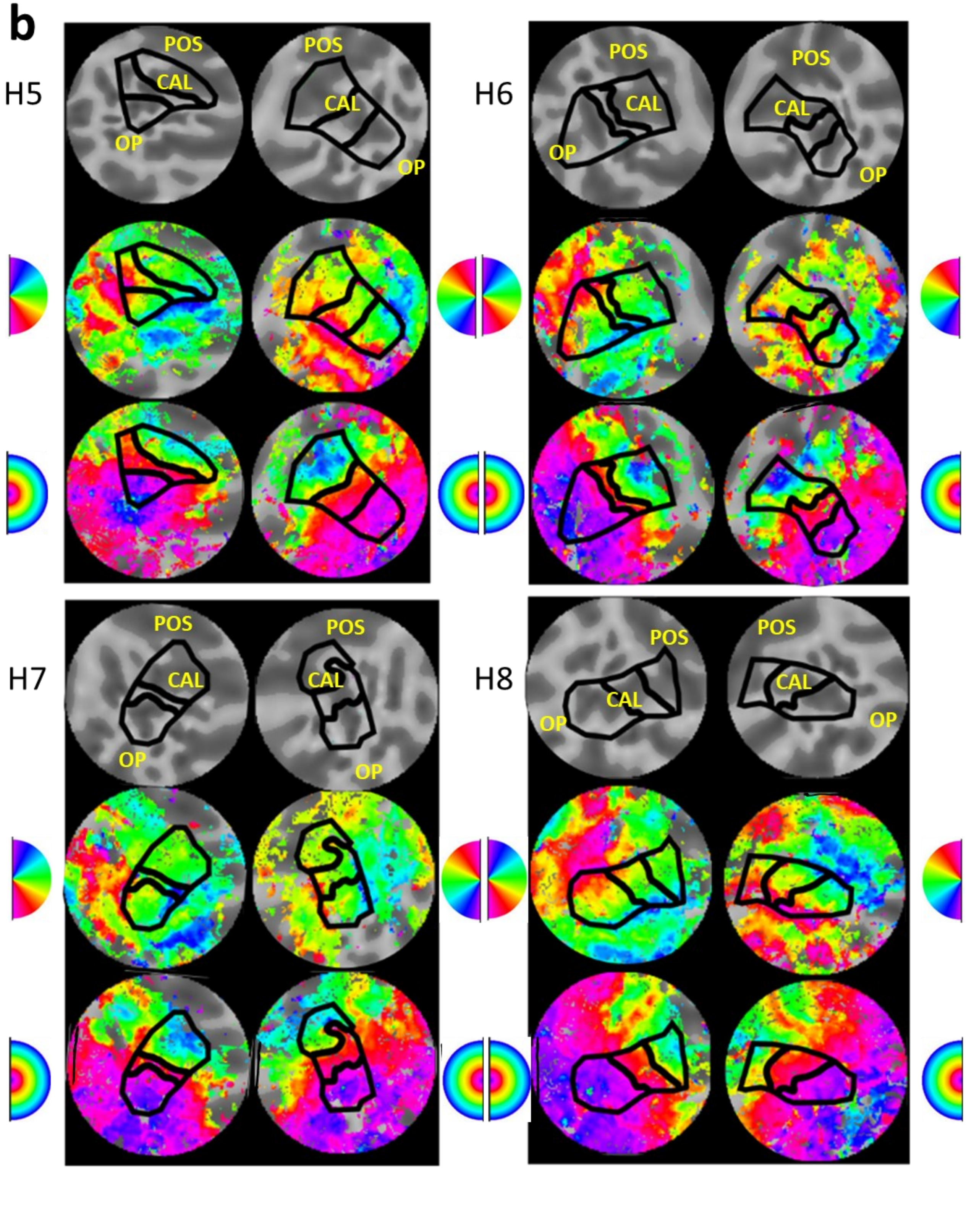
b
H5
POS
CAL
OP
POS
CAL
OP
H6
POS
CAL
OP
POS
CAL
OP
H7
POS
CAL
OP
POS
CAL
OP
H8
POS
CAL
OP
POS
CAL
OP

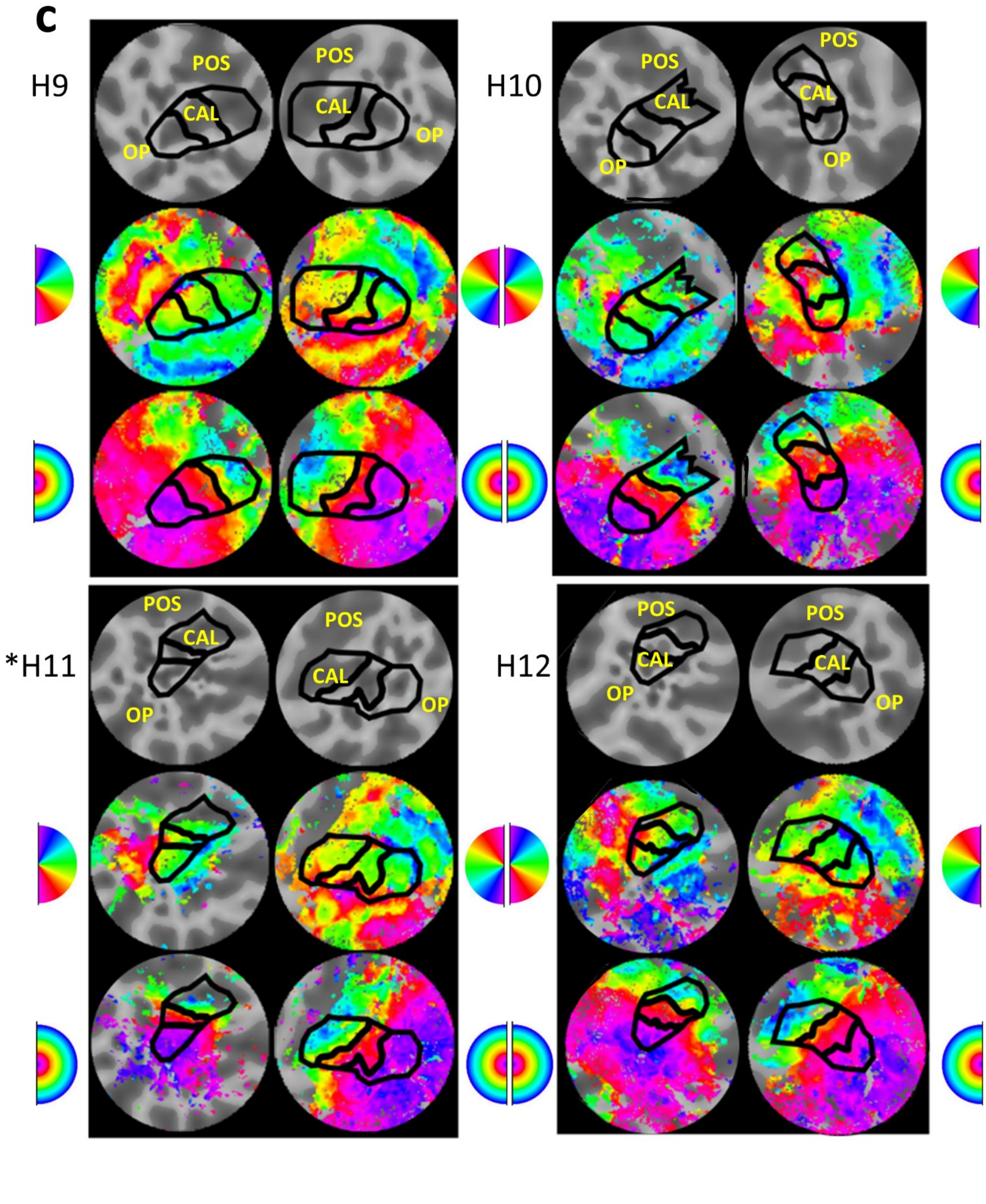
c
H9
POS
CAL
OP
POS
CAL
OP
H10
POS
CAL
OP
POS
CAL
OP
*H11
POS
CAL
OP
POS
CAL
OP
H12
POS
CAL
OP
POS
CAL
OP

d

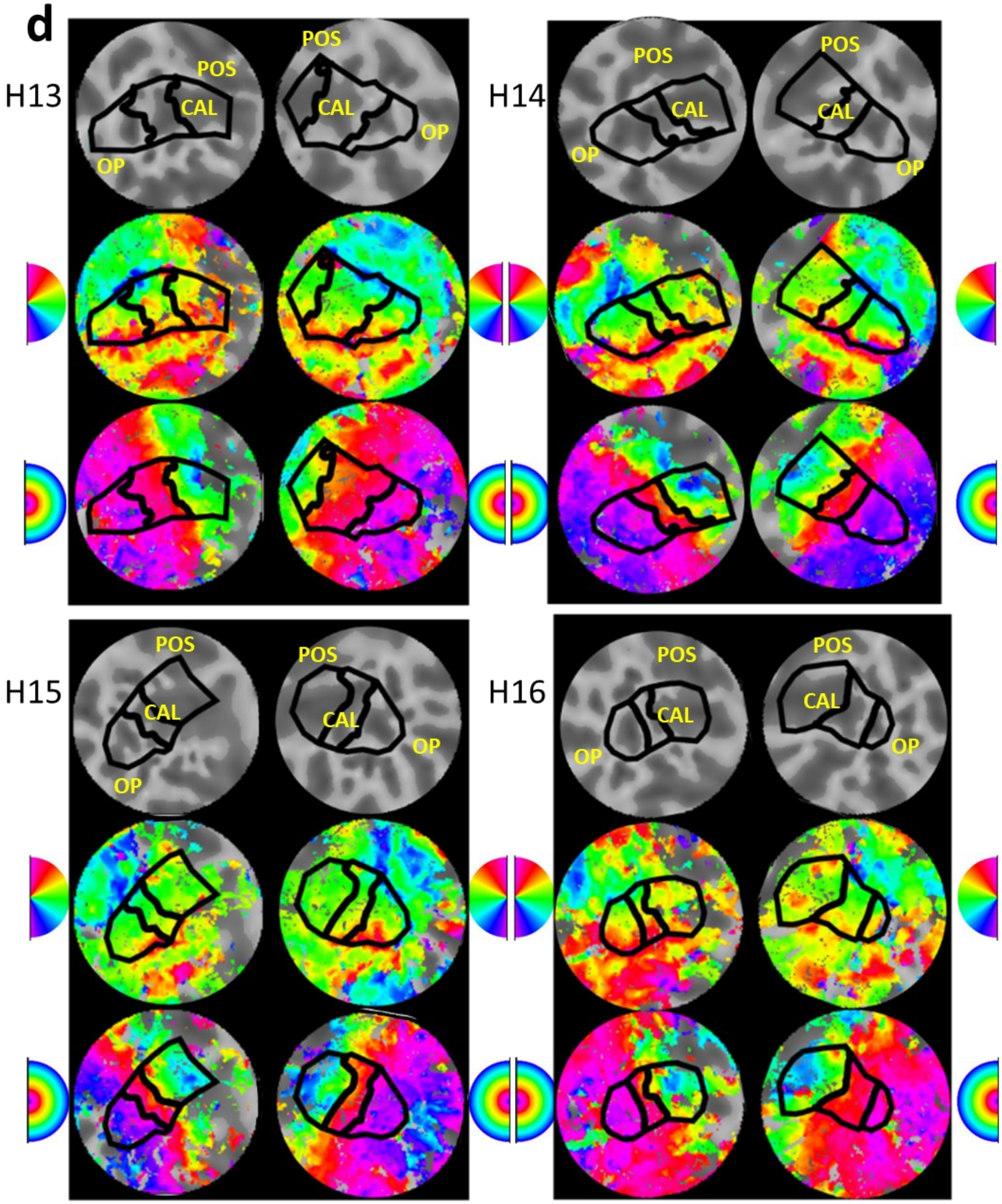
H13
POS
CAL
OP
POS
CAL
OP
H14
POS
CAL
OP
POS
CAL
OP
H15
POS
CAL
OP
POS
CAL
OP
H16
POS
CAL
OP
POS
CAL
OP

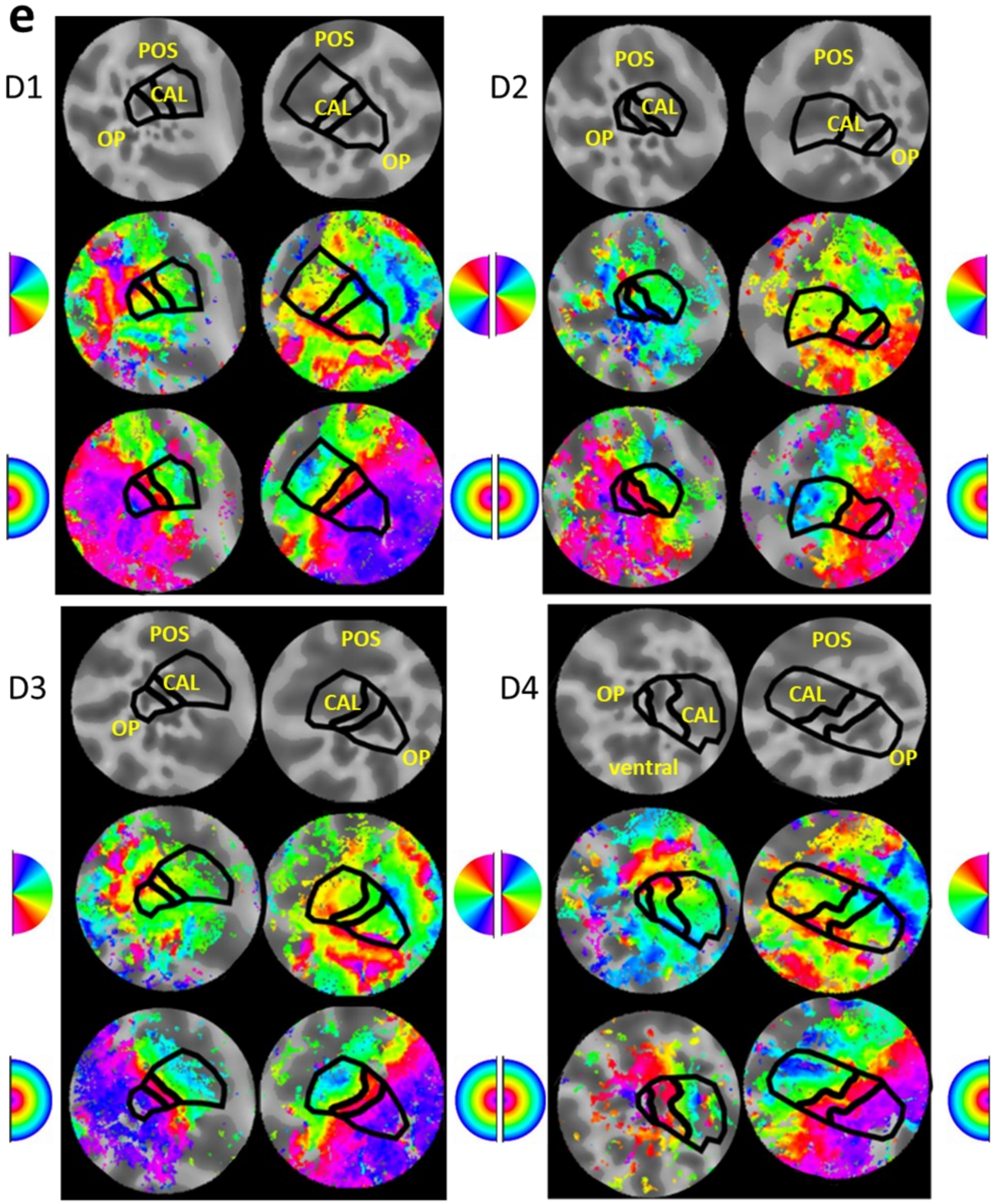
e
D1
POS
CAL
OP
POS
CAL
OP
D2
POS
CAL
OP
POS
CAL
OP
D3
POS
CAL
OP
POS
CAL
OP
D4
OP
CAL
ventral
POS
CAL
OP

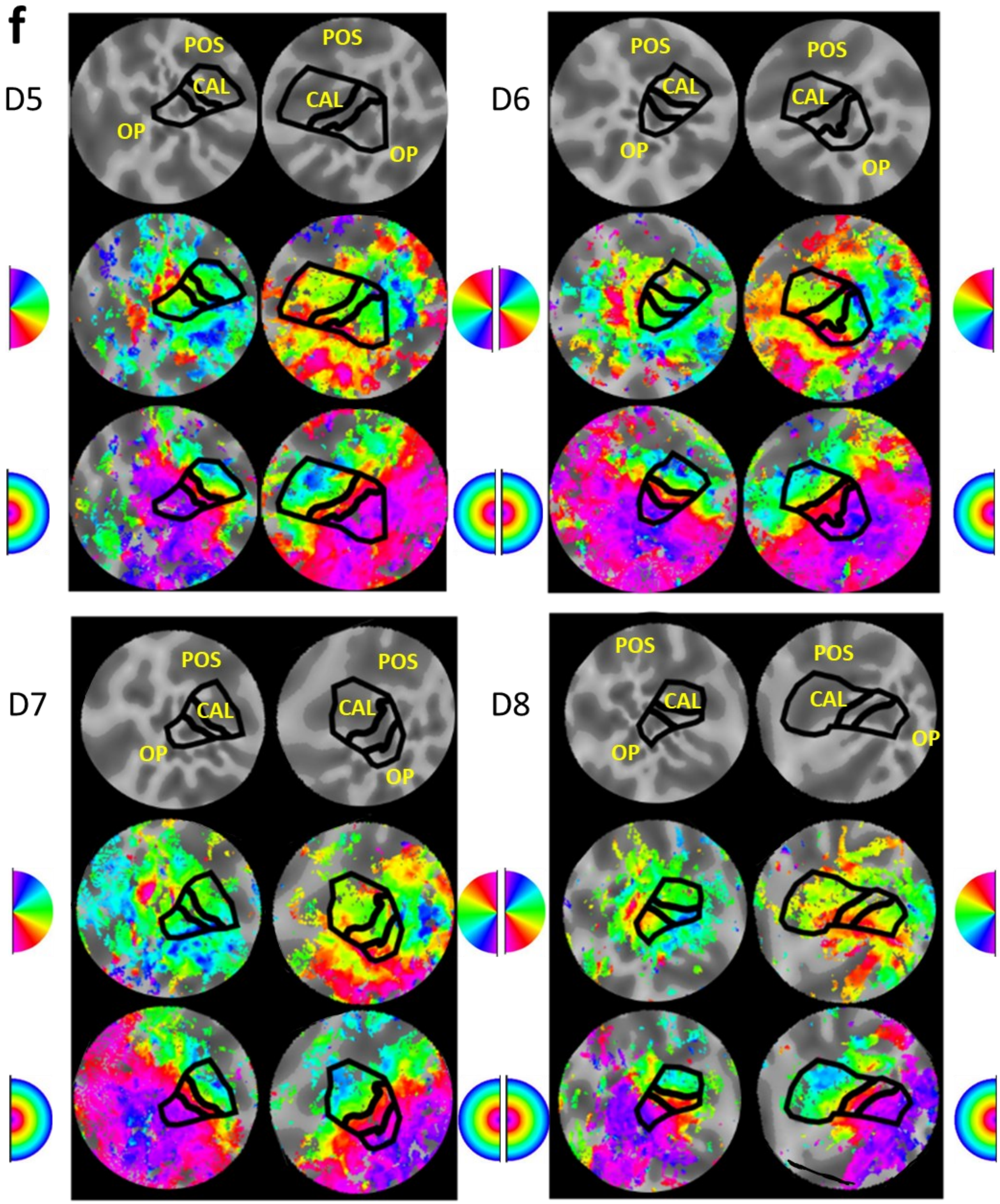
f
D5
POS
CAL
OP
POS
CAL
OP
D6
POS
CAL
OP
POS
CAL
OP
D7
POS
CAL
OP
POS
CAL
OP
D8
POS
CAL
OP
POS
CAL
OP

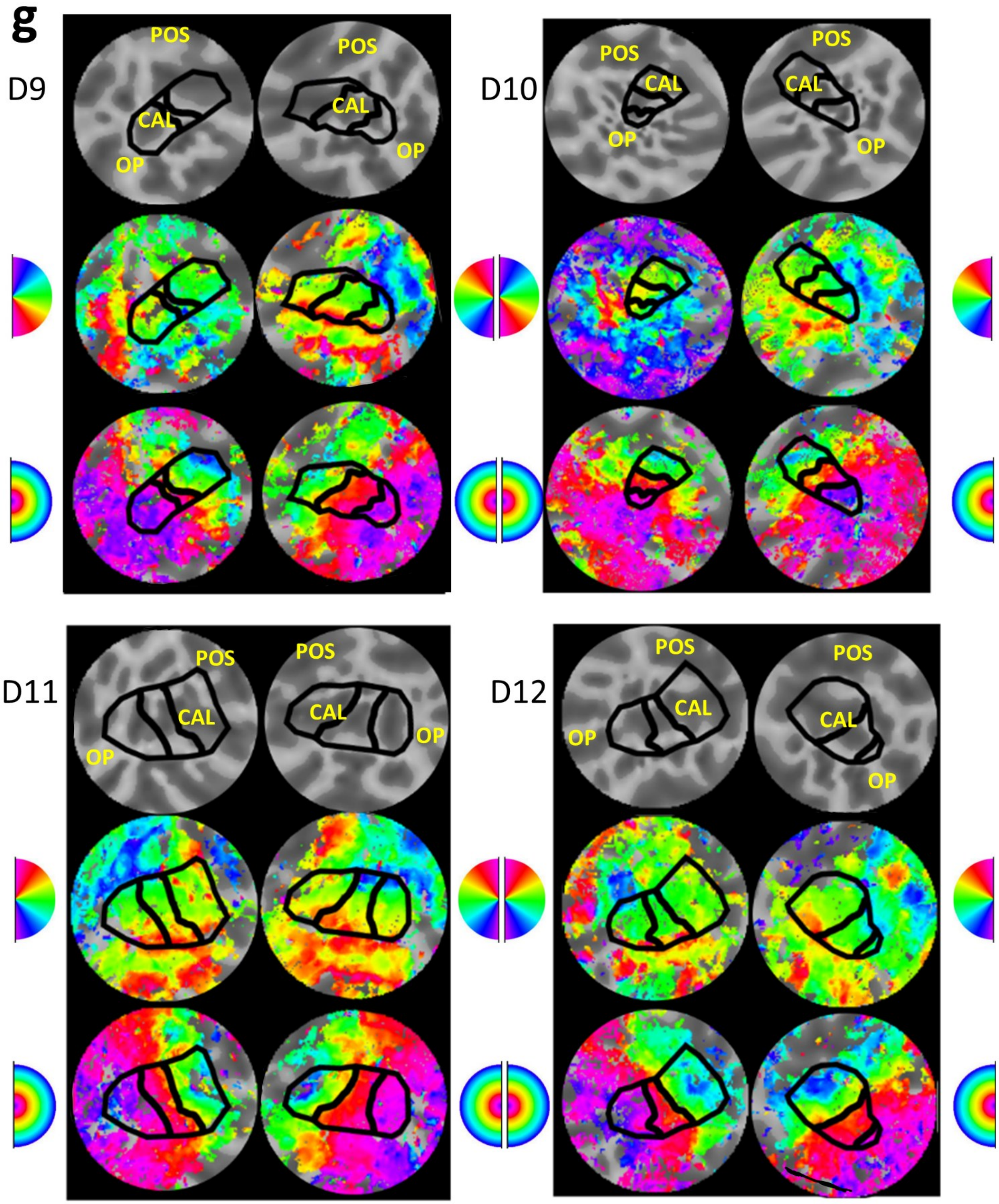
g
D9
POS
CAL
OP
POS
CAL
OP
D10
POS
CAL
OP
POS
CAL
OP
D11
POS
CAL
OP
POS
CAL
OP
D12
POS
CAL
OP
POS
CAL
OP

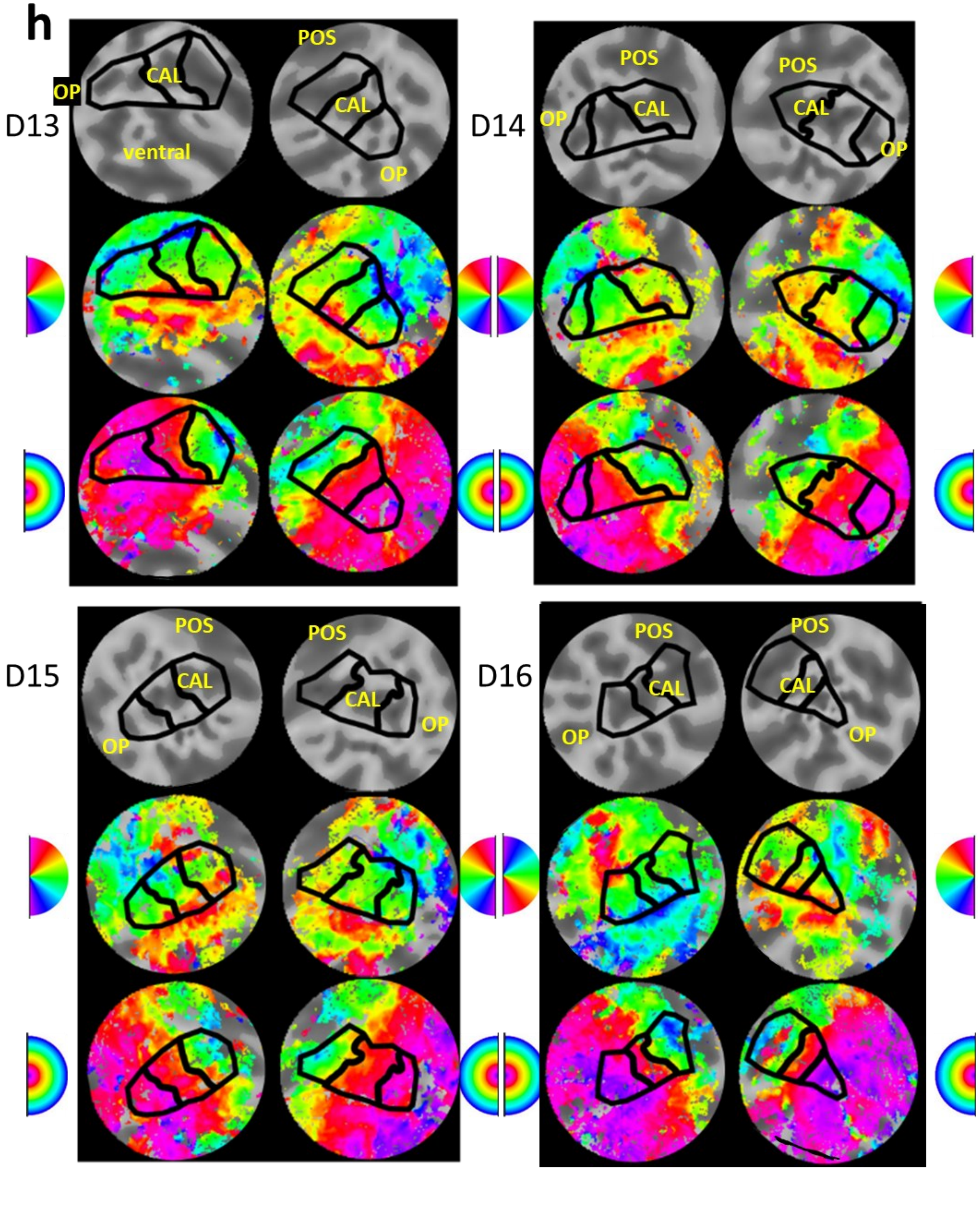
h
D13
D14
D15
D16
POS
CAL
OP
ventral

***Note regarding participant H11:** To maintain consistency across datasets, a standard coherence threshold of 0.20 was applied to the retinotopic maps. However, in the left hemisphere of participant H11, the far peripheral representation appeared abnormally small due to noisier data below this threshold compared to the right hemisphere. To accurately define this region of interest in H11, the boundaries were extended based on several converging lines of evidence. Firstly, a motion stimulus scan (moving dots) performed in the same session revealed robust activity exceeding the 0.20 threshold in the far periphery, and reducing the coherence threshold to 0.10 in the retinotopic mapping responses confirmed underlying activity in the area. Secondly, these functional markers were further verified using anatomical landmarks, ensuring the ROI followed the calcarine sulcus symmetrically across the upper and lower gyri, making it more comparable in size to the far peripheral representation in the right hemisphere. Details are shown in Supporting Information, Fig. S2i. Notably, as H11 is a hearing participant, any potential over-estimation of this ROI size would provide a conservative bias, as reducing its dimensions would only further amplify the reported finding of significantly larger peripheral representations in the D/deaf group compared to the hearing group.

Fig. S2(i). Details for hearing participant H11, showing justification for V1 sub-region boundaries in left hemisphere flat maps. Top left: Flattened surface without functional data. Gray scale represents anatomical surface curvature, with light gray denoting gyri and dark gray sulci. POS: Parieto-occipital sulcus; CAL: Calcarine sulcus; OP: Occipital pole. Top right: Polar angle map (rotating wedge stimulus) at two different coherence thresholds (> 0.10 and > 0.20). Middle right: Eccentricity map (expanding ring stimulus) at two different coherence thresholds (> 0.10 and > 0.20). Sample stimuli and color map legends shown to the left of each set of images. Bottom left: Moving dot stimulus paradigm consisting different types of motion (coherent radial or angular motion or incoherent motion alternating with blank screens in a block design (9 sec on, 9 sec off). Red cross indicates fixation point to place stimulus in the right hemifield, spanning 72 deg visual angle horizontally. Bottom right: Flat map showing regions responding in phase with the motion stimulus in yellow/orange and out of phase in all other colors, at two different coherence thresholds (> 0.10 and > 0.20). V1 boundaries and divisions between the three sub-regions are outlined in black. All other details as in Fig. S2(a-h).

i H11: Left hemisphere flat maps

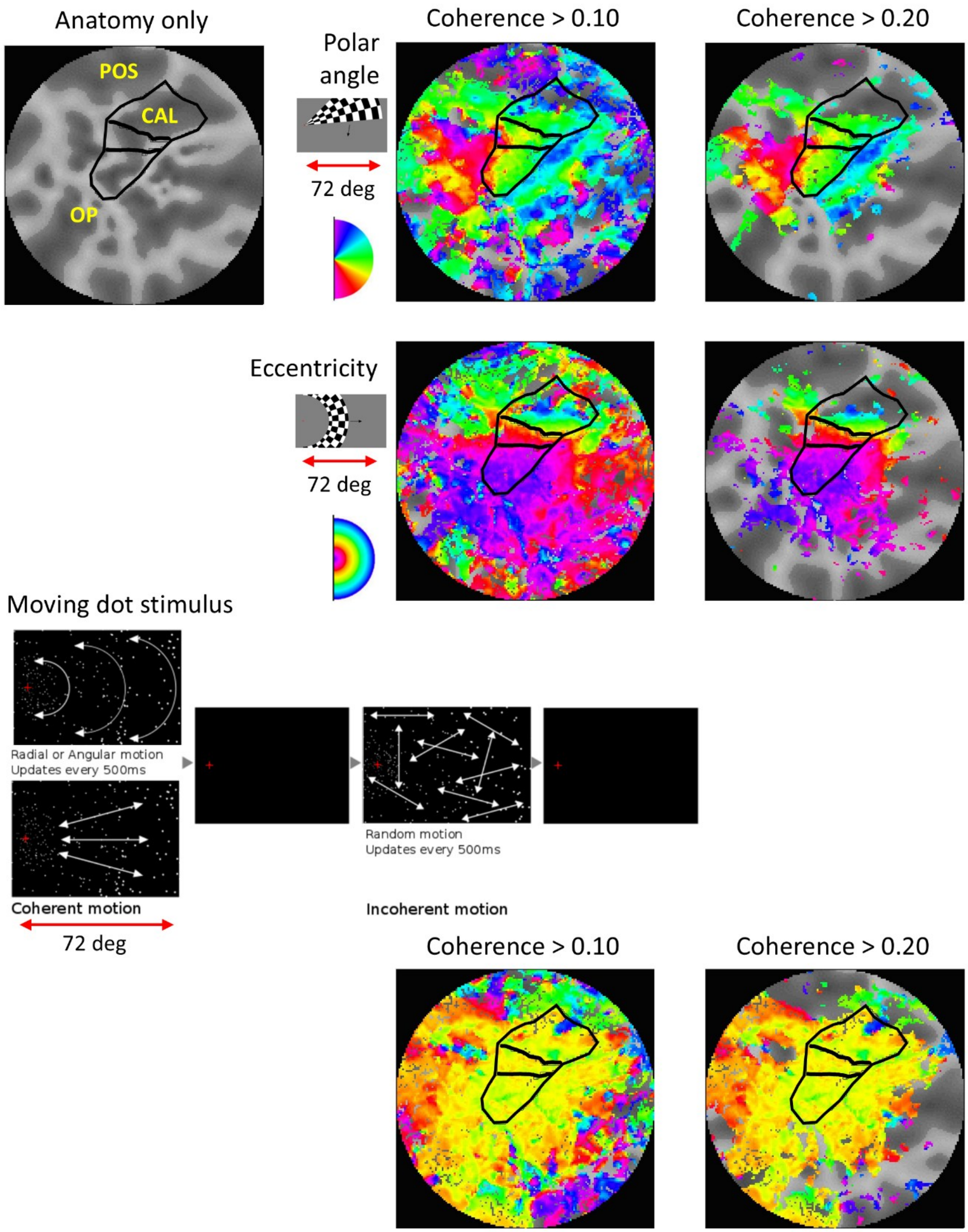

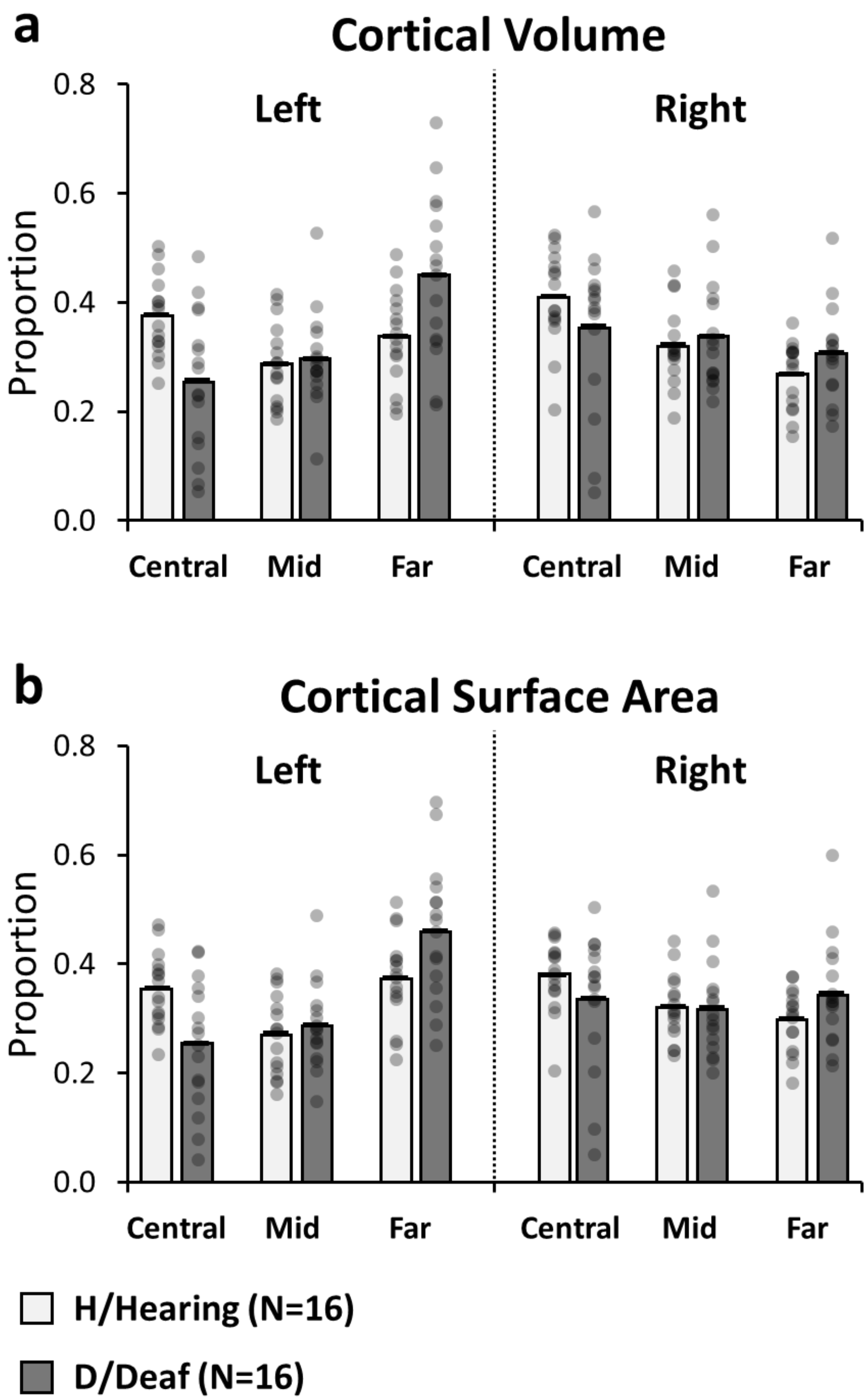

Fig. S3. Hemispheric differences in V1. (a) Cortical volume proportions and (b) Cortical surface area proportions for the D/deaf and hearing groups, divided into left and right hemispheres in sub-regions representing the Central (0-15°), Mid-peripheral (Mid, 15-39°) and Far-peripheral (Far, 39-72°) visual field. Dots represent individual participant data and bars represent the mean for each group. (Details as in Fig. 4b and d in the main text).

Dataset S1 (separate file). Microsoft Excel spreadsheet with anonymized data for all individual participants that went into producing all graphs in the manuscript and Supporting Information.

SI References:

J. H. Su, *et al.*, Thalamus Optimized Multi Atlas Segmentation (THOMAS): fast, fully automated segmentation of thalamic nuclei from structural MRI. *NeuroImage* **194**, 272–282 (2019).
